# Lsh Mediated RNA Polymerase II Stalling at HoxC6 and HoxC8 Involves DNA Methylation

**DOI:** 10.1371/journal.pone.0009163

**Published:** 2010-02-11

**Authors:** Yongguang Tao, Sichuan Xi, Victorino Briones, Kathrin Muegge

**Affiliations:** Laboratory of Cancer Prevention, SAIC-Frederick, National Cancer Institute, Frederick, Maryland, United States of America; Oregon State University, United States of America

## Abstract

DNA cytosine methylation is an important epigenetic mechanism that is involved in transcriptional silencing of developmental genes. Several molecular pathways have been described that interfere with Pol II initiation, but at individual genes the molecular mechanism of repression remains uncertain. Here, we study the molecular mechanism of transcriptional regulation at Hox genes in dependence of the epigenetic regulator Lsh that controls CpG methylation at selected Hox genes. Wild type cells show a nucleosomal deprived region around the transcriptional start site at methylated Hox genes and mediate gene silencing via Pol II stalling. Hypomethylation in *Lsh−/−* cells is associated with efficient transcriptional elongation and splicing, in part mediated by the chromodomain protein Chd1. Dynamic modulation of DNA methylation in *Lsh−/−* and wild type cells demonstrates that catalytically active DNA methyltransferase activity is required for Pol II stalling. Taken together, the data suggests that DNA methylation can be compatible with Pol II binding at selected genes and Pol II stalling can act as alternate mechanism to explain transcriptional silencing associated with DNA methylation.

## Introduction

DNA cytosine methylation is one of the major mechanisms involved in epigenetics since the pattern of DNA methylation is faithfully inherited by the next cell generation [1]. DNA cytosine methylation mediates transcriptional silencing and is crucial for the process of X inactivation, genomic imprinting and the control of developmental gene expression [2], [3]. Since DNA cytosine methylation is tightly linked with tumor suppressor gene silencing in cancer cells, it is also thought to play a role in tumorigenesis. The prevalent cytosine methylation in mammals occurs in the CpG context in differentiated cells, however, a recent study reports DNA methylation in a non-CpG context in human embryonal stem cells, possibly a unique feature associated with pluripotency [4].

CpG methylation shows an inverse relationship with transcript levels, and multiple mechanisms have been implicated to explain gene silencing in dependence of DNA methylation [1]. Although an effect of DNA methylation at the elongation phase of transcription on reporter transgenes has been reported [5], [6], most molecular mechanisms proposed to act on endogenous genes focus on the initiation phase of transcription. For example, DNA methylation can prevent binding of some transcriptional activators to their specific DNA sequences; furthermore, methyl-DNA binding proteins can recruit repressor complexes thus interfering with transcriptional initiation; moreover, DNA methyltransferases can interact with histone deacetylases rendering histones hypoacetylated. Histone acetylation in turn is part of the chromatin context that can facilitate the assembly of the Pol II and general transcription factor complex thus promoting transcriptional initiation [7]. In addition, a recent study demonstrated nucleosomal positioning at the transcriptional start site of the human MLH1 promoter in dependence of DNA methylation thus precluding Pol II binding [8]. These diverse mechanisms can interfere with transcriptional initiation, and for this reason, DNA methylation studies are frequently focused on promoter regions.

CpG methylation pattern are established and maintained by a family of DNA methyltransferases (Dnmt): Dnmt3a and Dnmt3b can act as *de novo* methyltransferases whereas Dnmt1 primarily maintains methylation pattern at the replication fork [1], [9]. In addition, other factors have been identified that support the establishment of DNA methylation pattern during development [9], for example NP95 enhances Dnmt1 association to hemi-methylated DNA, and the SNF2 chromatin remodeling family members ATRX controls methylation at ribosomal DNA and some satellite sequences. Lsh (Lymphoid specific helicase, also known as Pasg, HELLS, SMARCA6) is a SNF2 family member that controls DNA methylation during mammalian development. Likewise, the close A. thaliana homologue DDM1 (decrease in DNA methylation 1) affects DNA methylation in plants [10], [11], [12], [13]. Lsh can interact with Dnmts, and part of its effect on DNA methylation may be explained by enhanced association of Dnmts with genomic targets in the presence of Lsh [14], [15], [16], [17]. Among the genomic targets of Lsh are repeat elements, such as minor and major satellite sequences, Line and Sine elements and endogenous retroviral elements [10], [13], [18], [19]. In addition, several specific genomic sites were identified as targets for Lsh, such as p57, Pu.1, and pluripotency genes, Oct4 or Nanog [14], [17], [20], [21].

Previously, we reported that Lsh regulates the DNA methylation pattern and gene expression level of HoxA6 and HoxA7 genes in embryonic tissues and embryonal fibroblast lines [16]. Hox proteins are important developmental regulators that are controlled by tri-thorax group proteins, such as MLL (mixed lymphocyte leukemia), and by polycomb proteins [22]. Whereas MLL catalyzes methylation of histone H3 lysine 4 (H3K4me), the polycomb repressor complexes mediate H3K27 methylation and H2A ubiquitylation. In addition, a functional link between Dnmts and polycomb proteins has been identified [23], and it is now well documented that DNA methylation occurs during normal cellular differentiation at Hox gene clusters [24], [25], [26], [27]. Although DNA methylation is associated with Hox gene transcript levels, the mechanism of gene silencing remains uncertain, since polycomb mediated repression shows widespread Pol II stalling in *Drosophila* [28], but DNA methylation is thought to prevent Pol II binding and initiation [1].

To address the molecular mechanism of Hox gene silencing in dependence of Lsh and DNA methylation we examined nucleosomal positioning, RNA Pol II binding, RNA splicing and diverse histone modifications comparing wild type cells to hypomethylated *Lsh−/−* cells, or dynamically regulating DNA methylation levels in WT and *Lsh−/−* cells. Despite substantial differences in DNA methylation at HoxC genes we found similar nucleosomal positioning and abundant RNA Pol II binding in both *Lsh−/−* and wild type cells. Whereas methylated Hox genes in wild type cells show Pol II stalling, hypomethylated Hox genes have successful transcriptional elongation and efficient splicing. Moreover, dynamic modulation of DNA methylation in WT and *Lsh−/−* cells demonstrates a role for catalytically active Dnmt3b. Thus, our data suggests Pol II stalling as an alternate molecular pathway to induce transcriptional silencing at methylated genes.

## Results

**Figure 1:**
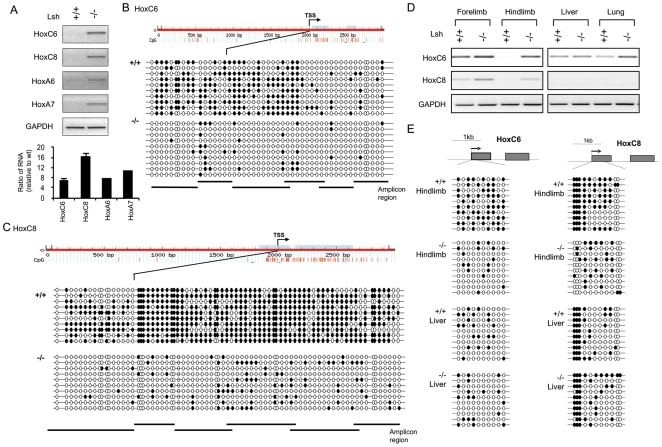
DNA methylation at silenced HoxC6 and HoxC8 genes. (A) RT-PCR analysis for detection of indicated Hox genes using total RNA derived from *Lsh+/+* and *Lsh−/−* MEFs (Top panel) and Real-time PCR analysis (Bottom panel). Primers were employed in exon1 and exon2 thus detecting spliced transcripts. Gene expression levels were normalized against the housekeeping gene Gapdh and are represented as fold increase in *Lsh−/−* cells compared to *Lsh+/+*. Error bars represent the mean standard deviation of three independent experiments. (B, C) Genomic DNA derived from *Lsh+/+* and *Lsh−/−* MEFs was examined by bisulphite sequencing at regions 2000 bp upstream and 1000 bp downstream of TSS at the HoxC6 (B) or HoxC8 (C) genes. Fifty of 53 CpG sites at the HoxC6 gene and 76 of 80 CpG sites at the HoxC8 gene were analyzed. Methylated CpGs are presented by black circles and unmethylated sites by open circles. The position of CpG sites is indicated by red lines in the graph and the light blue color indicates CpG islands according to http://www.urogene.org/methprimer/ (D) RT-PCR analysis for detection of indicated Hox genes in different tissues from *Lsh+/+* and *Lsh−/−* embryos d13.5. (E). Genomic DNA derived from indicated tissues from *Lsh+/+* and *Lsh−/−* embryos d13.5 was examined by bisulphite sequencing at regions indicated.

To study the molecular mechanisms of Lsh mediated gene silencing, we first examined Hox gene expression in murine embryonal fibroblast (MEF) cell lines derived from *Lsh−/−* and wild type (WT) embryos by RT-PCR analysis and Real-time PCR analysis (Fig. 1A). Similarly to our previous report on HoxA6 and HoxA7 genes, HoxC6 and HoxC8 mRNA expression was evident in *Lsh−/−* MEFs and repressed in WT MEFs [16]. Using chromatin immunoprecipitation (ChIP) analysis and specific antibodies against Lsh, we found that Lsh was associated with upstream and downstream regions of HoxC6, HoxC8 and HoxA6 genes (Fig. S1A,B,C). This suggests that Lsh, at least in part, acts directly on Hox gene expression. Similarly to our previous findings at HoxA6 and HoxA7 genes [16], we detected profound DNA methylation differences at the promoter regions of HoxC6 and C8 genes using methylation sensitive restriction enzymes followed by PCR analysis (Fig. S2A,B) and MeDIP analysis (Fig. S2C,D). To generate a precise CpG methylation map, bisulphite sequencing analysis was performed evaluating a region of about 2000 bp upstream and 1000 bp downstream of the TSS at either HoxC6 or HoxC8 genes (Fig. 1B,C). The overall DNA methylation levels varied greatly between WT cells and *Lsh−/−* samples. In particular, the methylation level at the CpG islands in close proximity to the TSS was reduced at HoxC6 (10 fold) and HoxC8 (7 fold) genes in *Lsh−/−* MEFs. This demonstrates that Lsh regulates DNA methylation at HoxC6 and HoxC8 genes in WT MEFs.

To address the question whether Lsh controls HoxC6 and HoxC8 genes during embryonic development [29], [30], we examined HoxC6 and HoxC8 gene expression by RT-PCR analysis in diverse tissues derived from day13.5 embryos. Forelimb, hindlimb and lung tissue showed signs of de-repression at HoxC6 and HoxC8 genes in the absence of Lsh, but HoxC8 expression in liver tissue was not affected by Lsh (Fig. 1D) suggesting that depletion of Lsh cannot re-activate specific Hox genes in every tissue, as has been previously described [16]. Bisulphite sequencing showed reduced DNA methylation in *Lsh−/−* hindlimb tissue compared to WT at HoxC6 and HoxC8 genes, similarly to the results in MEFs (Fig. 1E). Consistent with transcript levels, DNA methylation in liver tissue was not significantly changed comparing WT and *Lsh−/−* samples. Importantly, DNA hypomethylation was found at sites of active transcription, (HoxC6 in liver or *Lsh−/−* hindlimb), whereas DNA methylation was present at repressed sites (HoxC8 liver or WT hindlimb), supporting the idea that DNA methylation is closely associated with gene silencing. Overall, Lsh is associated with DNA methylation and is involved in gene silencing at HoxC6 and HoxC8 genes in embryo derived cell lines and in embryonal tissue at specific developmental stages.

**Figure 2:**
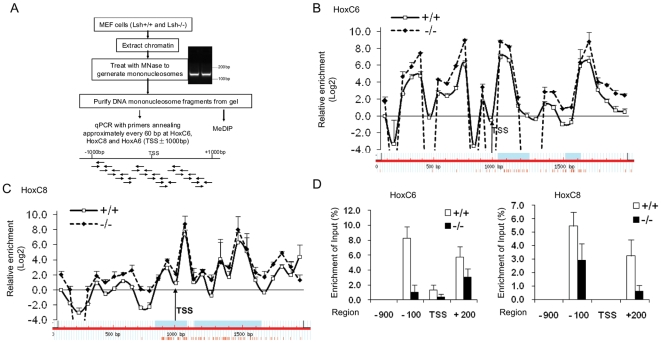
MNase protected sites are similar at methylated and hypomethylated Hox genes. (A) Diagram depicting the procedure for the MNase assay and the PCR amplicons used for the detection of Hox genes. The chromatin derived from *Lsh+/+* and *Lsh−/−* MEFs was treated with MNase. The DNA was then gel-purified and fragments less than 200 bp were used for real-time PCR. The amplicons were about 100±5 bp in size and were spaced 60±5 bp apart. The MNase protected profile of HoxC6(B) and HoxC8(C) genes was determined by normalizing the amount of the MNase derived PCR products to those of untreated DNA. Error bars represent the mean standard deviation of three to four independent experiments. (D) MeDIP analysis to enrich for methylated DNA at HoxC6 (left) and HoxC8 (right) genes of *Lsh+/+* and *Lsh−/−*samples after MNase treatment.

Phased nucleosomal positioning at TSS or enhancer elements is associated with active transcription, and regular nucleosomal pattern have been found at Hox clusters [31], [32], [33], [34]. Furthermore, Lsh is a homologue of SNF2 factors and may affect nucleosomal positioning at HoxC genes; although Lsh has not been demonstrated, as yet, to posses nucleosomal remodeling activity. To examine nucleosomal positioning at Hox genes, we performed a MNase protection assay [33] comparing mononucleosomes prepared from wild type or *Lsh−/−* samples by tiling PCR analysis (Fig. 2A). As shown in Fig. 2B,C, a similar pattern of MNase sensitivity around the promoter regions of HoxC6 and HoxC8 was observed comparing WT and *Lsh−/−* samples (for HoxA6 see Fig. S3A). The pattern suggested a fixed +1 nucleosome with a less pronounced −1 nucleosome and revealed a MNase unprotected region around the TSS. In further support, mononucleosomal DNA was subjected to MeDIP analysis to enrich for methylated DNA around the TSS of WT cells. Wild types samples revealed a MNase unprotected region around the TSS (Fig. 2D). Taken together, the results suggested that DNA hyper- or hypomethylation in WT or *Lsh−/−* cells had no detectable effect on nucleosomal positioning at those genes and wild type samples revealed a MNase unprotected region around the TSS similar to that in *Lsh−/−* samples.

**Figure 3:**
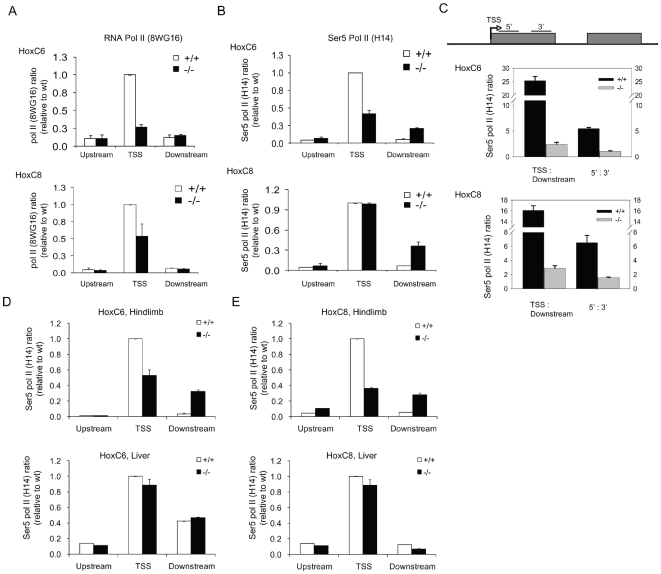
Stalling of RNA polymerase II at Hox genes. (A) Enrichment of unphosphorylated Pol II at HoxC6 (top) and HoxC8 (bottom) genes using ChIPs. Primers for upstream and downstream regions were positioned within 1000 bp of TSS (five primer sets for each group). TSS primers (two primer sets) covered the promoter-proximal region. The enrichment of unphosphorylated RNA Pol II (8WG16) binding in *Lsh+/+* and *Lsh−/−* MEFs was determined and normalized for better comparison to the binding at TSS in *Lsh+/+* (the value of the TSS region was set to 1). (B) Binding of Ser 5 Pol II (H14) using ChIPs at HoxC6 (top), and HoxC8 (bottom) at upstream, downstream regions and the TSS region as in (A). (C) Ratios of Ser 5 Pol II ChIP signals for the TSS region to the downstream region and the ratios of Ser 5 Pol II signal using a primer set located 5′ and 3′ of exon1. Error bars represent the standard deviation for the mean of three independent experiments. (D,E) Binding of Ser 5 Pol II (H14) at HoxC6 (D), and HoxC8 (E) at upstream, downstream regions and the TSS region in hindlimb (top) and liver tissues (bottom).

Nucleosomal deprived regions can be associated with active promoter regions and engaged RNA Pol II [33]. Thus, we tested the idea whether the MNase unprotected region around the TSS of Hox genes in WT or *Lsh−/−* samples could be linked to Pol II binding. First, we confirmed the position of the major TSS sites at either HoxC6 and HoxC8 genes by using 5′RACE and site specific RT-PCR analysis (Fig. S4). Then, ChIPs analysis was performed using specific antibodies raised against the heptapeptide repeat of the carboxyterminal domain (CTD) of Pol II. Chromatin samples derived from *Lsh−/−* MEFs showed enrichment of Pol II at the TSS of HoxC6 and HoxC8 genes (Fig. 3A, and for HoxA6 Fig. S3B). Likewise, WT samples showed a peak of unphosphorylated Pol II binding at TSS consistent with the hypothesis that Pol II binding may interfere with nucleosomal occupancy and may determine phasing of the +1 nucleosome [33]. These findings suggest that CpG island methylation at HoxC6 and HoxC8 genes does not inevitably hinder Pol II binding. Moreover, repression of HoxC6 and HoxC8 transcription was not simply caused by a lack of Pol II binding but by a molecular mechanism following Pol II engagement.

Recent results of genome-wide analysis reported RNA Pol II occupancy at promoter regions of genes that were thought to be transcriptional silent [35], [36]. At those promoters a Pol II initiation complex is assembled but the elongation process is repressed leading to Pol II stalling. To test for Pol II stalling in transcriptional repressed WT MEFs ChIPs analysis was performed for detection of Ser5 phosphorylated Pol II which is associated with transcription initiation and early elongation or promoter clearance [37]. The binding of Ser5 Pol II at the promoter region was compared to the binding at the gene body to assess successful Pol II elongation. Whereas WT and *Lsh−/−* samples showed binding of Ser5 Pol II at the TSS of HoxC6 and HoxC8 (Fig. 3B and for HoxA6 Fig. S3B), only *Lsh−/−* samples were enriched for Ser5 Pol II at the gene body consistent with the hypothesis of successful elongation in the absence of Lsh. The ratio of Pol II binding at the TSS over the gene body (downstream) determines the Pol II stalling index [38]. The Pol II stalling index was comparatively high in WT samples (about 16 to 25) and low in *Lsh−/−* samples (2 to 3 fold)(Fig. 3C). In further support, the ratio of Ser5 Pol II binding to a 5′region over a 3′ region at the first exon at either HoxC6 or HoxC8 genes was 4 to 6 fold higher in WT samples compared to *Lsh−/−* samples (Fig. 3C and for HoxA6 Fig. S3C). Furthermore, to address the question whether Pol II stalling could be detected in WT embryonic tissue, ChIPs analysis for detection of Ser 5 Pol II was performed for hindlimb and liver tissue comparing WT and *Lsh−/−* embryos. Wild type hindlimb samples showed exclusive enrichment for Ser5 Pol II binding at the TSS region, but not at the gene body, suggesting Pol II stalling. In contrast, *Lsh−/−* hindlimb samples showed a similar Pol II enrichment at the TSS region and the gene body (Fig. 3D) indicating successful Pol II elongation. Liver samples revealed no significant differences between WT and *Lsh−/−* samples. In particular, HoxC6 showed a high level of Ser5 Pol II engagement at TSS and gene body, whereas HoxC8 had only enrichment at the TSS site consistent with the observation of transcript levels in those tissues. Taken together, a Pol II initiation complex can engage in *Lsh−/−* and WT cells, at hyper or hypomethylated TSS regions; however, Pol II binding at the gene body is only associated with hypomethylated HoxC6 and HoxC8 genes. Thus, the observation of Pol II stalling versus Pol II elongation is closely associated with the pattern of DNA methylation at HoxC6 and HoxC8 genes.

**Figure 4:**
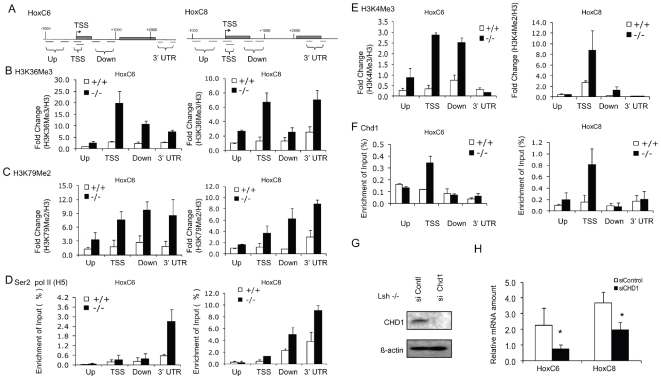
Chromatin marks associated with transcriptional elongation are increased in the absence of Lsh. (A) Schematic diagram depicting the position of the primer pairs (black lines) used for ChIP analysis. Comparison of the ratio of H3K36Me3 (B), H3K79Me2 (C) and H3K4Me3 (E) enrichment to total H3 occupancies (Fig. S6) between Lsh WT and *Lsh−/−*MEFs at up, TSS, down and 3′UTR regions of HoxC6 and HoxC8 genes. (D) ChIP analysis for the detection of Ser 2 RNA Pol II (H5). (F) ChIP analysis for detection of Chd1. (G) Western analysis for detection of Chd1 protein comparing whole cell extracts derived from *Lsh−/−*MEFs targeted by Chd1 siRNA or control siRNA (H) Real-time RT-PCR analysis detecting HoxC6 and HoxC8 mRNA in *Lsh−/−* MEFs after targeting by Chd1 siRNA compared to control treated cells. Results represent standard deviations for the mean of three independent experiments. Asterix indicates a p value p<0.05.

To support further the notion of Pol II stalling versus elongation, histone modifications that are hallmarks of RNA Pol II elongation and enriched at the gene body were assessed [35], [39], [40]. Histone 3 lysine 36 trimethylation (H3K36me3) and H3K79 dimethylation (H3K79me2) are present at fairly equal levels in cellular extracts of *Lsh−/−* and WT cells (Fig. S5). Both H3K36me3 and H3K79me2 showed enrichment at the gene body of HoxC6 and HoxC8 genes in *Lsh−/−* cells compared to WT cells (Fig. 4A, B, C). Furthermore, Ser2 Pol II enrichment at the 3′ end, another hallmark of successful transcriptional elongation [36] was increased at HoxC6 and HoxC8 genes in *Lsh−/−* cells compared to WT samples (Fig. 4D). Taken together, the change in histone modifications and Ser2 Pol II further supported the observation of Pol II stalling at HoxC6 and HoxC8 genes in WT cells and successful Pol II elongation and transcription in *Lsh−/−* MEFs.

To reveal part of the molecular mechanism that is involved in transcriptional elongation in *Lsh−/−* cells, we examined H3K4 trimethylation (H3K4me3) and binding of Chd1. The chromodomain protein Chd1 recognizes the lysine methyl mark and has been shown to promote transcriptional elongation [41], [42]. Previously, we reported increased H3K4 methylation levels at hypomethylated repeat elements in *Lsh−/−* cells and 5-Azacytidine treated WT cells [43]. As shown in Fig. 4E, *Lsh−/−* samples had increased of H3K4me3 at HoxC6 and HoxC8 genes, whereas WT samples showed very little enrichment. In addition, Chd1 bound more efficiently at HoxC6 and HoxC8 genes in *Lsh−/−* samples compared to WT samples (Fig. 4F). The difference in Chd1 association may be due to HoxC specific differences in H3K4me3 levels or due to slightly higher overall Chd1 protein levels in *Lsh−/−* cells (Fig. S5). To test whether Chd1 is functionally involved in HoxC6 and HoxC8 gene expression, *Lsh−/−* cells were specifically depleted of Chd1 mRNA (Fig. S6B) and Chd1 protein (Fig. 4G) using small interfering RNA (siRNA). Within 48 hrs of siChd1 RNA treatment, HoxC6 and HoxC8 mRNA showed a significant suppression (46% and 67% respectively) compared to control siRNA treated *Lsh−/−* cells (Fig. 4H). This suggests that Chd1 plays a functional role in the transcription of HoxC6 and HoxC8 genes in *Lsh−/−* cells.

**Figure 5:**
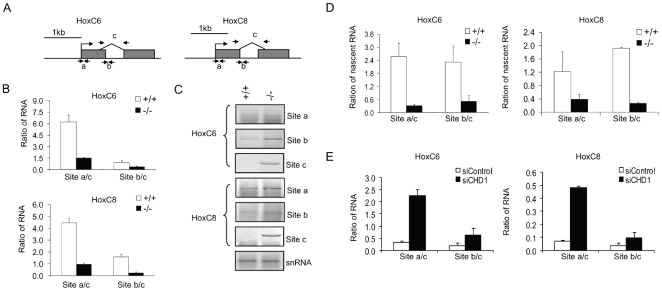
Enhanced transcriptional elongation and generation of spliced mature transcripts after Lsh depletion. (A) Schematic representation of the probe and primer pairs used to detect 5′ primary transcripts, unspliced transcripts and spliced mRNA. (B) Real time RT-PCR analysis detecting the ratio of unspliced to spliced mRNA of the HoxC6 and HoxC8 gene from *Lsh+/+* and *Lsh−/−* MEFs. Results represent the mean and standard deviations of three independent experiments. (C) RT-PCR analysis for detection of HoxC6 and HoxC8 transcripts derived from nuclei of *Lsh+/+* and *Lsh−/−* MEFs. U2 small nuclear RNA (snRNA) was unaffected by Lsh and served as a control. (D) Real-time RT-PCR analysis from four independent experiments to detect the ratio of unspliced to spliced nascent transcripts of HoxC6 and HoxC8 genes by nuclear run on assay. (E) Real time RT-PCR analyses detecting the ratio of unspliced to spliced mRNA of the HoxC6 and HoxC8 genes in *Lsh−/−* MEFs after Chd1 siRNA treatment. Error bars represent the standard deviations for the mean of three independent experiments.

To delineate further the role of Chd1 in transcription, we examined the maturation of pre-mRNA to spliced RNA since Chd1 associates with elongation factors as well as splicing components, and consequently, depletion of Chd1 impairs the splicing efficiency [41], [42]. Primers were designed to detect transcripts at HoxC6 and HoxC8 genes: for detection of ‘total’ transcripts (at the first exon,a), for pre-mRNA (at the exon/intron boundary,b), or for spliced RNA (c, spanning both exons)(Fig. 5A). The ratio of total transcripts to spliced RNA (a/c) and pre-mRNA to spliced RNA (b/c) was lower in *Lsh−/−* samples compared to WT samples suggesting that *Lsh−/−* cells were relatively more efficient in splicing than WT cells (Fig. 5B). Nuclear RNA samples derived from WT cells showed some evidence of transcripts close to the TSS (site a) and transcripts of pre-mRNA (site b) but barely detectable spliced transcripts (site c) in contrast to *Lsh−/−* samples (Fig. 5C). To rule out effects of RNA long-term stability and to quantify transcription rates, a nuclear run-on assay was performed using Br-UTP labeling for detection of nascent RNA [44]. Efficient immunoprecipitation was detected in cells that had been labeled with Br-UTP indicating the specificity of the assay (Fig. S6A,B). Using nascent transcripts, the ratio of pre-mRNA to spliced transcripts was less than one in *Lsh−/−* samples indicating efficient splicing (Fig. 5D). In contrast, WT samples showed a 3 to 8 fold higher ratio in relation to *Lsh−/−* when either total transcripts to mature mRNA (a/c) or pre-mRNA to spliced mRNA (b/c) were compared. Therefore, the data suggests that WT samples compared to *Lsh−/−* samples are impaired in converting HoxC6 and HoxC8 pre-mRNA templates into mature transcripts. To further support the notion that Chd1 is involved in splicing in *Lsh−/−* cells, we examined Chd1 depleted *Lsh−/−* cells (as shown in Fig. 4G). Upon Chd1 depletion, the ratio of pre-mRNA to spliced transcripts reverted back to ratios similar to those in WT samples pointing to a functional role for Chd1 in splicing in (Fig. 5E). Taken together, the data suggests an inadequacy in transcriptional elongation and generation of spliced mature RNA in WT cells, and successful elongation and splicing of pre-mRNA in Lsh−/− cells that involves Chd1.

**Figure 6:**
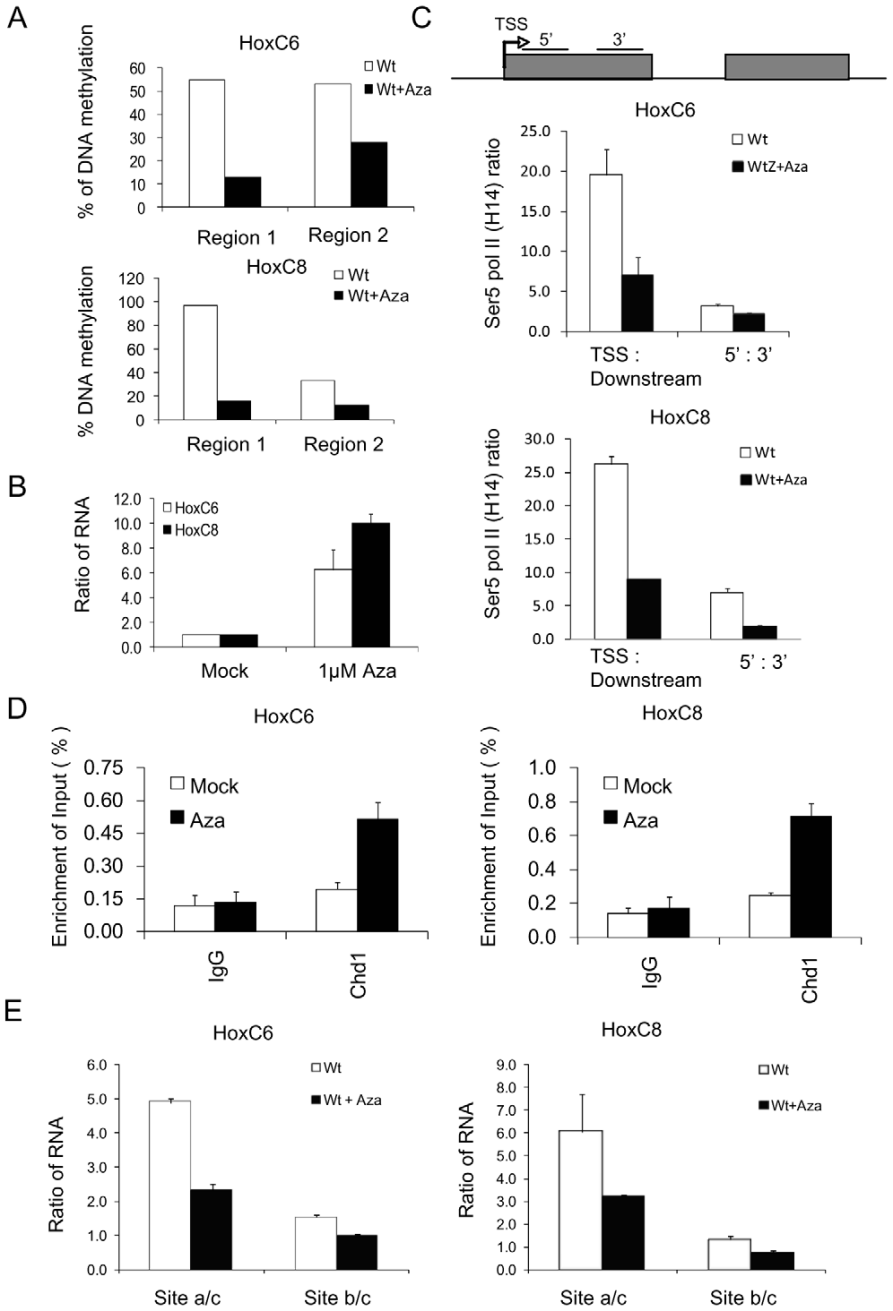
5-Azacytidine treatment overcomes Pol II stalling. (A) Summary of bisulphite sequencing results derived from ten clones (Figure S8) to illustrate the percentage of reduced CpG methylation after 5-Azacytidine treatment at the first 10 (HoxC6, Up) or 11 (HoxC8, Down) CpG sites downstream of TSS (region 1) or 10 (HoxC6) or 12 (HoxC8) CpG sites at the second exon (region 2) (B) Real-time PCR analysis for detection of HoxC6 and HoxC8 genes in *Lsh+/+* MEFs before and after five days of 5-Azacytidine (Aza) treatment (1 µM). The mock treated samples were set to one for better comparison. (C) Ratio of Ser 5 Pol II ChIP signals of the TSS region over the downstream region and the ratio of 5′r and 3′regions of exon1 comparing chromatin derived from *Lsh +/+* MEFs of mock and 5-Azacytidine treated cells. (D) ChIPs analysis for detection of Chd1 in the TSS region comparing chromatin derived from WT MEFs of mock and 5-Azacytidine treated cells. IgG serves as a negative control. (E) Real time RT-PCR analyses detecting the ratio of unspliced to spliced mRNA of the HoxC6 and HoxC8 gene from *Lsh+/+* and *Lsh−/−* MEFs after the treatment of 5-Azacytidine.

To address the question, whether changes in DNA methylation by other means than Lsh depletion can affect Pol II stalling, we treated WT MEFs with 5-Azacytidine. Bisulphite sequencing indicated reduced CpG methylation at the TSS region (region1) and further downstream at the second exon comprising the splicing signal (region2) after 5-Azacytidine treatment (Fig. 6A and Fig. S7). Also, hypomethylated WT cells showed re-activation of repressed HoxC6 and HoxC8 transcripts (Fig. 6B). Moreover, the Pol II stalling index changed 3 to 4 fold at HoxC6 and HoxC8 genes after 5-Azacytidine treatment (Fig. 6C). In addition, ChIPs analysis revealed an increase of Chd1 binding at HoxC6 and the HoxC8 genes after 5-Azacytidine treatment, similar to enhanced Chd1 binding observed in *Lsh−/−*samples (Fig. 6D). Finally, the ratio of total RNA to spliced transcripts (a/c) and pre-mRNA to spliced transcripts (b/c) were reduced after treatment of WT cells with 5-Azacytidine suggesting an improved conversion of pre-mRNA into mature transcripts similar to that seen in *Lsh−/−* samples (Fig. 6E). Taken together, the data suggested that 5-Azacytidine treatment can overcame Pol II stalling in WT cells.

**Figure 7:**
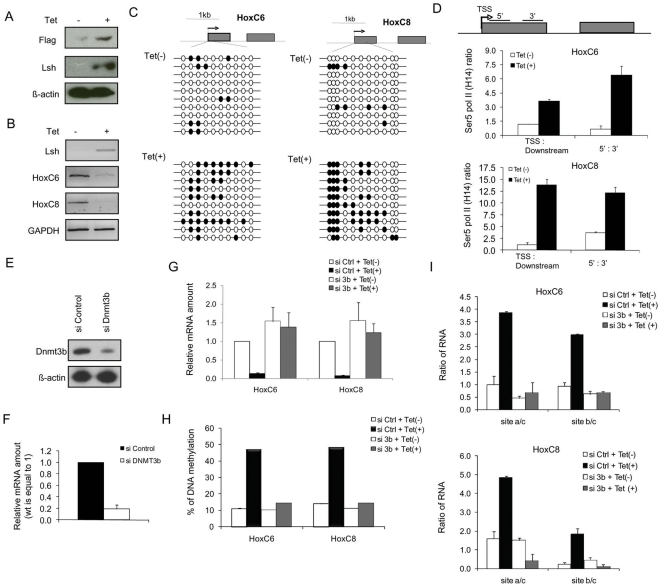
Re-introduction of Lsh into *Lsh−/−* cells restores Pol II stalling and requires the presence of Dnmt3b. (A) Western analysis using anti-Lsh antibodies or anti-Flag antibodies to detect Lsh protein expression in Lsh−/− MEFs stably transfected with the tet-off repressor Lsh vector before and after 24 hrs of tetracycline treatment. Note, that traces of Lsh protein were visible after longer exposure of the blot indicating a slight leakiness of the repressor system. (B) RT-PCR analysis for detection of HoxC6 and HoxC8 and Lsh mRNA comparing extracts derived from *Lsh−/−*MEFs before and after 24 hours of tetracycline treatment. (C) Genomic DNA derived from *Lsh−/−* MEFs (before and after 24 hrs of tetracycline treatment) was examined by bisulphite sequencing at regions downstream of TSS at the HoxC6 (Left) and the HoxC8 (Right) genes. (D) Ratio of Ser 5 Pol II ChIP signals for the TSS region to the downstream region and the ratio of Ser 5 Pol II signal using a primer set located 5′ and 3′ of exon1 comparing chromatin derived from *Lsh−/−* MEFs before and after 24 hours of tetracycline treatment to induce Lsh. Error bars represented the standard errors of the mean of three independent experiments. Western Blot (E) and Real-time RT-PCR analysis (F) for detection of Dnmt3b mRNA from Lsh inducible cells treated with control siRNA (siControl) or siRNA targeted against Dnmt3b (siDnmt3b) for 48 hrs. (G) Real time RT-PCR analyses detecting the ratio of unspliced to spliced mRNA of the HoxC6 and HoxC8 gene in *Lsh−/−* MEFs after targeting by Dnmt3b siRNA or control siRNA before and after 24 hours of tetracycline treatment to induce Lsh. (H) Summary of bisulphite sequencing results derived from ten clones for each sample (Figure S10) to illustrate the percentage of CpG methylation loss at the first 10 (HoxC6, left) or 10 (HoxC8, right) CpG sites downstream of TSS in Lsh−/− MEFs after targeting by Dnmt3b siRNA or control siRNA before and after 24 hours of tetracycline treatment to induce Lsh. (I) Real time RT-PCR analyses detecting the ratio of unspliced to spliced mRNA of the HoxC6 and HoxC8 genes in *Lsh−/−* MEFs after targeting by Dnmt3b siRNA or control siRNA before and after 24 hours of tetracycline treatment to induce Lsh.

To understand whether dynamic regulation of DNA methylation in MEFs can cause Pol II stalling in hypomethylated Lsh−/− cells, we attempted to restore Lsh function. Lsh was introduced into *Lsh−/−* MEFs using an inducible tetracycline dependent expression system. As shown in Fig. 7A, Lsh protein levels were enhanced within 24 hrs of tetracycline treatment. Also, HoxC6 and HoxC8 mRNA expression was reduced after Lsh induction, as demonstrated by RT-PCR analysis (Fig. 7B). This change in gene expression was accompanied by some gain in CpG methylation at the TSS regions of HoxC6 and HoxC8 genes (from 11 to 35% at HoxC6 and from 8 to 47% for HoxC8) and at the second exon next to the splicing signal (Fig. 7C and Fig. S8A,B). This result was consistent with our previous observations that Lsh can directly associate with Hox genes (Fig. S1) and that Lsh is required for targeting of Dnmt3b to HoxA genes [16]. Moreover, Lsh re-expression in *Lsh−/−* cells increased the Pol II stalling index (Fig. 7D), further supporting a close connection of Lsh mediated DNA methylation and Pol II stalling.

To demonstrate that restoration of Pol II stalling does require Dnmt3b we performed Dnmt3b depletion (Fig. 7E,F) by siRNA interference in *Lsh−/−* cells that expressed tetracycline inducible Lsh. As shown in Fig. 7G, only control siRNA treated *Lsh−/−*cells showed HoxC6 and HoxC8 gene suppression after Lsh induction, whereas Dnmt3b depleted cells failed to do so. Bisulphite sequencing and methylation sensitive PCR analysis indicated increased CpG methylation after Lsh induction when Dnmt3b was present, but not in Dnmt3b depleted cells (Fig. 7H and Fig. S9 Fig. S10). This suggests that Dnmt3b plays a role in Lsh mediated DNA methylation at those sites. In addition, only control siRNA treated *Lsh−/−* cells showed a shift in the ratio of pre-mRNA to spliced transcripts towards the WT phenotype after tetracycline induced Lsh expression, but not the Dnmt3b depleted *Lsh−/−* cells (Fig. 7I). Thus, Lsh function is not simply ‘associated’ with DNA methylation levels, but does indeed require Dnmt3b to mediate gene silencing and Pol II stalling at Hox genes.

**Figure 8:**
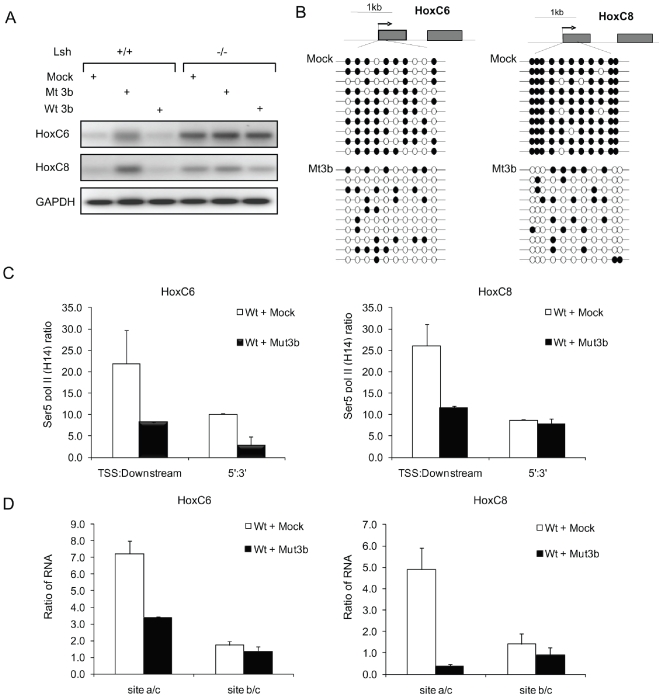
The catalytic domain of Dnmt3b is required for Pol II stalling. (A) RT-PCR analysis for the detection of HoxC6 and HoxC8 mRNA from *Lsh+/+* and *Lsh−/−* MEFs transfected with wild type Dnmt3b (Wt 3b) and Mutant Dnmt3b (Mut 3b) for 72 hrs. (B) Bisulphite sequencing results to illustrate reduced CpG methylation at the first 10 (HoxC6, Left) or (HoxC8, Right) in samples prepared from WT MEF cells transfected with Mut 3b for 72 hrs. (C) Ratio of Ser 5 Pol II ChIP signals for the TSS region over the downstream region and the ratio of Ser 5 Pol II signal using a primer set located 5′ and 3′ of exon1 comparing chromatin from WT MEFs after the transfection with Mut 3b. (D) Real time RT-PCR analysis detecting the ratio of unspliced to spliced mRNA of the HoxC6 (left) and HoxC8 (right) gene in WT MEFs after the transfection with Mut 3b.

Finally, we examined the role of catalytically active Dnmt3b in HoxC6 and HoxC8 gene silencing. We transfected WT MEFs with a mutant form of Dnmt3b (a kind gift of Dr Hsieh) that had been previously shown to be catalytically inactive [45]. RT-PCR analysis showed an increase in HoxC6 and HoxC8 mRNA in WT cells that had been transfected with mutant Dnmt3b, in contrast to control WT cells (mock transfected or transfected with wild type Dnmt3b) (Fig. 8A). Bisulphite sequencing indicated effective reduction of CpG methylation at HoxC6 and HoxC8 TSS regions after introduction of mutant Dnmt3b (Fig. 8B). Calculation of the Pol II stalling index showed that transfection of mutant Dnmt3b reduced Pol II stalling in WT cells at HoxC6 and HoxC8 genes (Fig. 8C). Furthermore, the ratio of pre-mRNA to spliced transcripts shifted towards a profile comparable to that generated by *Lsh−/−* deletion (Fig. 8D). Taken together, the data suggests that catalytically active Dnmt3b is functionally involved in Pol II stalling at HoxC6 and HoxC8 genes.

## Discussion

### Nucleosomal occupancy and DNA methylation at Hox genes

DNA methylation is a stable epigenetic mark that is generally associated with repressed, compact and less ‘accessible’ chromatin. Several studies that assessed nucleosomal distribution identified some genomic sites that were influenced by DNA methylation as well as sites that were unaffacted (reviewed in [46]). The effect of DNA methylation was enhancing as well as preventing nucleosomal occupancy (for example at the chicken beta globin locus) making it necessary to examine nucleosomal position at the individual promoter level [8], [46]. In the current study we found no evidence by MNase profiling that the level of DNA methylation (Fig. 1,2) influences the position of nucleosomes at HoxC6 and HoxC8 genes. While our MNase assay may be limited to determine subtle changes (less than 60 bp) in nucleosomal positioning, the comparable binding pattern of Pol II at TSS in WT and *Lsh−/−* samples further supports the notion of a similar nucleosomal organization that is independent of DNA methylation levels. As pointed out above, other genomic sites, for example the human bidirectional MLH1 promoter, shows nucleosome deposition in the context of DNA methylation, and loss of nucleosomal occupancy after Azacytidine treatment [8]. The specific DNA sequence, the relative degree and the precise pattern of CpG methylation, and the different cell types (human cancer cell versus MEF) may all affect the outcome. Also, the position of the CpG island may be important since an in vitro system demonstrated that CpG island sequences itself could have a destabilizing effect on nucleosome positioning analogue to the role of AT rich sequences in S cerevisiae [47]. Based on the many suggested molecular mechanisms, it is difficult to predict nucleosomal positioning, and it is therefore advisable to examine individual methylated promoter regions. Our study indicates in principle that DNA methylation at CpG islands can be compatible with accessibility and does not inevitably exclude Pol II engagement.

### Pol II stalling versus elongation

We identified here the mechanism of gene silencing, and found in ‘methylated’ WT cells Pol II stalling and in hypomethylated cells all the hallmarks of transcriptional elongation, including histone modifications and Ser2 Pol II transition and effective splicing. Pol II stalling was originally demonstrated to control the expression of heat shock genes and a number of proto-oncogenes, but recent reports suggest widespread regulation of Pol II elongation, including developmentally regulated Hox genes [28], [34], [36]. A genome wide study in human cells found Pol II promoter stalling in embryonic stem cells as well as somatic B cells and hepatocytes [35]. In murine macrophages, LPS inducible genes are in part regulated by transition of the pre-associated Ser5 phosphorylated Pol II into the elongation phase [48]. In addition, unstimulated genes showed low levels of pre-mRNA that were quickly degraded. After stimulation, Ser2 Pol II transition and full length mature transcripts were deteceted indicating that splicing was effectively regulated [48]. As for transcriptional regulation of Hox genes we provide evidence that Chd1 is involved, a chromodomain protein that associates with elongation as well as splicing factors. The inverse relationship of DNA methylation and H3K4me3 may serve as recruitment signal for Chd1 that recognizes directly the tri-methylation mark. Chd1 can bridge H3K4me3 with FACT or PAF that are engaged in transcriptional elongation [41], [42]. In addition, Chd1 physically associates with spliceosomal components (U2 snRNP subunits) [42]. Consequently, depletion of H3K4me levels (using siRNA interference of the HMT Ash2) results in reduced association of Chd1 to specific chromatin targets and a decrease in the recruitment of splicing components (SF3A1 and SF3A2) to selected genomic sites. Furthermore, depletion of splicing factors such as SC35 reduces transcriptional elongation suggesting a close relationship between elongation and splicing [44].

### The role of DNA methylation

We describe here that Pol II promoter stalling at HoxC6 and HoxC8 genes in WT cells depends on Lsh and is tightly associated with DNA methylation. There are multiple lines of evidence for a functional role of DNA methylation beyond the observation of Pol II stalling in WT cells and elongation in hypomethylated Lsh−/− cells 1. Lsh and Dnmt3b bind directly to Hox genes and Lsh regulates Dnmt3b binding [16]. 2. Dynamic regulation of DNA methylation in WT cells by 5-Azacytine treatment or modulating Dnmt3b activity overcomes Pol II stalling 3. Likewise, restoration of DNA methylation mediated by wild type Lsh in *Lsh−/−* cells restores Pol II stalling. 4. Restoration by Lsh requires the presence of Dnmt3b 5. Pol II stalling requires the catalytic site of Dnmt3b and thus requires DNA methylation. Though Pol II stalling at the HoxC6 and HoxC8 genes involves DNA methylation, clearly not all Pol II stalling is attributed to DNA methylation and Pol II stalling is found at unmethylated promoter regions [2]. For Hox genes there may be multiple levels of control of how DNA methylation contributes to Pol II stalling: for example, a site specific increase of H3K4me3 may follow reduced DNA methylation [6], [27], [43], [49], [50] and the level of subsequent Chd1 recruitment may influence elongation and splicing. Also, DNA methylation levels are involved in the modulation of polycomb protein binding and their subsequent histone modifications [16], [23]. The silencing effect of polycomb proteins has been linked to altered chromatin environment and Pol II stalling and, recently, it was proposed that a steric hindrance excluding Pol II elongation may mediate polycomb silencing [28]. Whether methylated CpG island genes, other than at HoxC6 and HoxC8 genes, can also show Pol II stalling requires further investigation. In particular, the position of CpG sites (upstream, around or downstream of TSS) deserves further attention since it had been shown that DNA methylation downstream of TSS can impact transcriptional elongation at integrated transgenes or episomal plasmids [5], [6]. A recent genome wide DNA methylation analysis observed a strong association of methylation peaks with the translational initiation codon [51]. Furthermore, a lack of DNA methylation peaks at translational initiation codons correlated well with Pol II elongation efficiency, consistent with the idea that DNA methylation can regulate Pol II elongation.

Lastly, aberrant DNA methylation patterns at tumor suppressor genes and at Hox genes have been found in cancer cells, but the mechanism of transcriptional silencing for many sites remains uncertain [16], [24], [26], [52], [53], [54], [55]. Our study provides evidence that DNA methylation at some TSS can be compatible with Pol II binding. Therefore Pol II stalling may serve as an alternate mechanism for gene silencing at some methylated tumor suppressor genes and may suggest the possibility of novel strategies to interfere with gene silencing in cancer cells.

## Materials and Methods

### Cell culture, tissues, plasmids and siRNA

The mouse embryonic fibroblasts (MEF) [56] were treated with 1 µM 5-Azacytidine (Sigma) for 5 days. Embryonic tissues were derived from embryos of day13.5 of gestation. Dnmt3b plasmids Wt3b (pMT3b) and Mut3b (pMt3bMut, that contains a single point mutation at position 657 converting cysteine to serine resulting in catalytically inactive enzyme) [45] were a kind gift from Dr. Hsieh (University of Southern California) The pT-REx-Lsh plasmid was constructed by subcloning Flag-tagged Lsh [57] into the pcDNA4/TO vector (Invitrogen). *Lsh−/−* MEFs were transfected with pcDNA6/TR (Invitrogen) and pT-Tex-Lsh and stable clones were selected with 300 µg/ml Zeocin and 10 µg/ml Blasticidin. Lsh protein was induced by adding 6 µg/ml tetracycline. For si RNA interference cells were transfected with Dnmt3b, Chd1siRNA or control siRNA (Dharmacon) using Dhamafect Duo Transfection Reagent.

### Western analysis and antibodies

Equal amounts of protein (20∼50 µg) were separated on 4–12% Bis-Tris SDS-PAGE (Invitrogen) and transferred onto a nitrocellulose membrane. The following antibodies were used: normal mouse or rabbit IgG (Upstate), rabbit anti-Lsh recombinant protein affinity-purified antibody [16]; 8WG16 Pol II (ab817), H14 Pol II (ab24759), H5 Pol II (ab24758, anti-H3 (ab1791), anti-H3 (di methyl K79) (ab3594), anti-H3 (tri methyl K36) (ab9050), anti-H3 (tri methyl K4) (ab8580) (Abcam); 5-Methylcytidine (BI-MECY-0100)(Eurogentec); anti-Flag (F1804), anti-β-actin (A2228), anti-Brdu (B2531)(Sigma); anti-Dnmt3b (ALX-804-233-C100)(Alexis Biochemicals); anti-CHD1 (NB100-60411) (Novus Biologicals Inc).

### PCR analysis

For RT-PCR analysis total RNA was extracted using Qiagen RNeasy Mini Kit and 2 µg were reverse transcribed with 50 ng random primers and 10U Reverse Transcriptase (Superscript III Kit, Invitrogen). The cDNA was diluted 1∶10, and 2 µl were used for analysis. Results were normalized for expression of the housekeeping gene Gapdh. For methylation-sensitive PCR analysis [58] DNA was extracted by using Qiagen DNeasy kit and 1.0 µg was digested with *Mae* II (Roche) or *BssH* I (NEB). PCR analysis was carried out as follows: 3 min at 94°C, 35 cycles of 30 s at 94°C, 30 s at 55°C, and 30 s at 72°C, and finally 5 min at 72°C. For Real-time PCR analsysi as described by [16] a standard curve was generated by serially diluting input samples. For MNase digestion the fold difference was calculated [59] between MNase-treated and untreated samples. Values used were collected from the linear range of amplification. Primers are listed in the supplemental Table S1.

### MNase protection analysis

The assay was carried out as descriped previously [60]. In brief, after lysis of single cell suspensions, chromatin was digested with MNase, DNA extracted by phenol:chloroform and separated on a 1.5% agarose gel. This procedure yielded ∼80% DNA from mononucleosome (∼150 bp) and ∼20% DNA from di- and trinucleosomes (∼300 and ∼450 bp, respectively). The mononucleosomal DNA was excised, gel-purified, and used as a PCR template.

### ChIP and MeDIP analysis

ChIPs were performed as previosuly described (Xi et al., 2007). Sonication yielded fragments between 300 to 500 bp in average. For detection of H3 modifications data was normalized by performing simultaneously ChIP analysis using anti-H3 antibodies. Before carrying out MeDIP, genomic DNA was digested with *Mse* I or/and *Pst* I to produce random fragments ranging in size from 200 to 500 bp. Oneµg of gel purified DNA was used for a MeDIP assay as described previously [2]. Real-time PCR analysis was performed, for each primer the amplication efficiency was calculated and the data expressed as enrichment related to Input.

### Bisulphite sequencing

Genomic DNA (1 µg) was subjected to bisulphite treatment by using MethylDetector Bisulphite Modification kit (Active Motif). Amplified fragments were subcloned into the pCR2.1-TOPO vector (Invitrogen). Independent clones were sequenced by using the M13 F primer and only sequences with individual fingerprint selected for analysis.

### Nuclear run-on assays

Nuclear RNA was extracted from the nuclei using RNeasy mini kit (Qiagen). The nuclear run-on assay has been previously described [44]. In brief, a NTP mix plus BrUTP was used wheras the control reactions for monitoring nonspecific binding omitted NTPs. The run-on reaction was performed at 25°C for 15 min on about 10^7^ permeabilized cells and DNase I–treated total RNA was extracted and immnuprecipitated using anti-BrU antibody (B2531, Sigma) andµl Protein G Dynabeads (Invitrogen). Recovered RNA was reverse transcribed and analyzed by Real-time PCR.

## Supporting Information

Figure S1 Lsh association with Hox genes. ChIP analysis using specific antibodies against Lsh (or control IgG) followed by PCR analysis to detect association of Lsh with promoter regions, TSS, and gene body regions of HoxC6 (A), HoxC8 (B) and HoxA6 (C) genes comparing Lsh+/+ and Lsh−/− MEFs. Enrichment is represented as a percentage of input. Error bars represent the standard deviation for the mean of three independent experiments.
(0.50 MB TIF)

Figure S2 DNA methylation analysis at HoxC6 and HoxC8 genes. (A,B) Methylation sensitive PCR to determine DNA methylation level at HoxC6 (A) and HoxC8 (B) genes. The map on top illustrates the position of methylation-sensitive restriction enzyme (Mae II), as well as the primers used for the methylation-sensitive PCR analysis (Top). PCR analysis using genomic DNA derived from Lsh+/+ and Lsh−/− MEFs after digestion with Mae II. The undigested DNA served as a control for equal input of DNA (Uncut). (C,D) MeDIP analysis for detection of CpG methylation at the HoxC6 (C) or HoxC8 (D) genes. Region 1 (devoid of CpG sites) was amplified as negative control. Genomic DNA was digested with Mse I (C) or Mse I and Pst I (D), immunoprecipitated with anti-5-methylcytosine antibodies and specific Hox locations assessed by real-time PCR. Binding was expressed as a percentage of the input DNA. The bar graphs represent the mean of two independent MeDIP experiments.
(0.39 MB TIF)

Figure S3 MNase protection profile at HoxA6. (A) The MNase protected profile of HoxA6 was determined by normalizing the amount of the MNase derived PCR products to those of the untreated DNA. (B) Enrichment of unphosphorylated Pol II (left) and Ser 5 Pol II (right) at HoxA6. (C) Ratio of Ser 5 Pol II ChIP signals at the TSS region over the downstream region of HoxA6 and the ratio of Ser 5 Pol II signal using a primer set located within exon1.
(0.43 MB TIF)

Figure S4 Identification of major transcriptional start sites (TSS) at HoxC6 and HoxC8 genes. (A). Identification of HoxC6 TSS using cDNA derived from Lsh−/− MEFs and primer specific for TSS region 1 and region 2. RT-PCR analysis suggest that regions 2 (as marked in Fig. 1 and Fig. 2 as TSS) is indeed the major TSS for HoxC6 since no signal was detected fro region 1. (B) Identification of HoxC8 TSS using cDNA derived from Lsh−/− MEFs and primer specific for TSS region 1. (C,D) Identification of HoxC6 (C) and HoxC8 (D) TSS using RLM-RACE. RNA is treated with tobacco acid pyrophosphatase (TAP) to remove the cap structure from the full-length mRNA leaving a 5′-monophosphate which is subsequently ligated to a synthetic RNA adaptor. After reverse transcription nested gene specific primers and adaptor specific primers are used for gene specific amplification. Results indicate a major amplification product about 320 bp for HoxC6 and 290 bp for HoxC8 confirming the preferential use of the predicted TSS for both genes. The major TSSs are reported in the Mouse Genome database (MGI) at chr15:102,839,993 for HoxC6 and at chr15:102,820,970 for HoxC8.
(0.63 MB TIF)

Figure S5 Protein analysis in the absence of Lsh. (A) Western analysis of extracts derived from Lsh+/+ and Lsh−/− MEFs using the indicated antibodies. (B) Real-time RT-PCR analysis for detection of Chd1 mRNA from Lsh−/− MEFs treated with control siRNA (siControl) or siRNA targeted against Chd1 (siChd1) for 48 hrs.
(0.46 MB TIF)

Figure S6 Nuclear run on assay. (A) Schematic diagram for the nuclear run-on assay based on the use of BrUTP. The nascent transcript snRNA detection was demonstrated by analyzing total RNA from Lsh+/+ and Lsh−/− MEFs labeled with BrU, Uridine (U) and BrU in the presence of α-amanitin. (B) PCR products with reverse transcriptase (+RT) or without reverse transcriptase (-RT) from nascent RNA that were immunoprecipitated by anti-Br-UTP antibody for detection of nascent RNA generated in the nuclear run on assay. The WT sample generated with RT served as positive control. Data indicate the purity of the nascent RNA used for the assay.
(0.43 MB TIF)

Figure S7 DNA hypomethylation after 5-Azacytidine treatment. Genomic DNA derived from Lsh+/+ MEFs before and after treatment of 5-Azacytidine was examined by bisulphite sequencing at region 1 (top) and region 2 (2nd exon, bottom) at the HoxC6 (A) and the HoxC8 (B) genes.
(0.72 MB TIF)

Figure S8 Re-introduction of Lsh in Lsh−/− MEFs. Genomic DNA derived from Lsh+/+ and Lsh−/− MEFs before and after 24 hours of tetracycline treatment to induce Lsh was examined by bisulphite sequencing at regions of the second exon at the HoxC6 (A) or HoxC8 (B) genes. Methylated CpGs are presented by black circles and unmethylated sites by open circles.
(0.74 MB TIF)

Figure S9 DNA hypomethylation after Dnmt3b depletion. Methylation sensitive PCR to determine methylation level at HoxC6 (A) and HoxC8 (B) after targeting by Dnmt3b siRNA or control siRNA before and after 24 hours of tetracycline treatment to induce Lsh in Lsh−/− cells. The map on top illustrates the position of methylation-sensitive restriction enzyme (Mae II in HoxC6 and BssH II), as well as the primers used for methylation-sensitive PCR analysis (Top). PCR analysis using genomic DNA derived from Lsh−/− MEFs after digestion with Mae II and BssH II. The digested DNA without enzyme sites served as a control for equal input of DNA (Bottom).
(0.11 MB TIF)

Figure S10 Dnmt3b is required for re-methylation at HoxC6 and HoxC8. Genomic DNA derived from Lsh−/− MEFs after targeting by Dnmt3b siRNA or control siRNA before and after 24 hours of tetracycline treatment to induce Lsh. HoxC6 (A) and the HoxC8 (B) genes were examined by bisulphite sequencing. Methylated CpGs are presented by black circles and unmethylated sites by open circles.
(1.39 MB TIF)

Table S1 Primers for PCR, ChIP, Nuclear Run on and real-time PCR
(0.16 MB DOC)
