# Supplementary figures and images for: Lsh Mediated RNA Polymerase II Stalling at HoxC6 and HoxC8 Involves DNA Methylation
Source: PLoS One. 2010 Feb 11;5(2):e9163. doi: 10.1371/journal.pone.0009163 (PMC2820093; doi:10.1371/journal.pone.0009163)

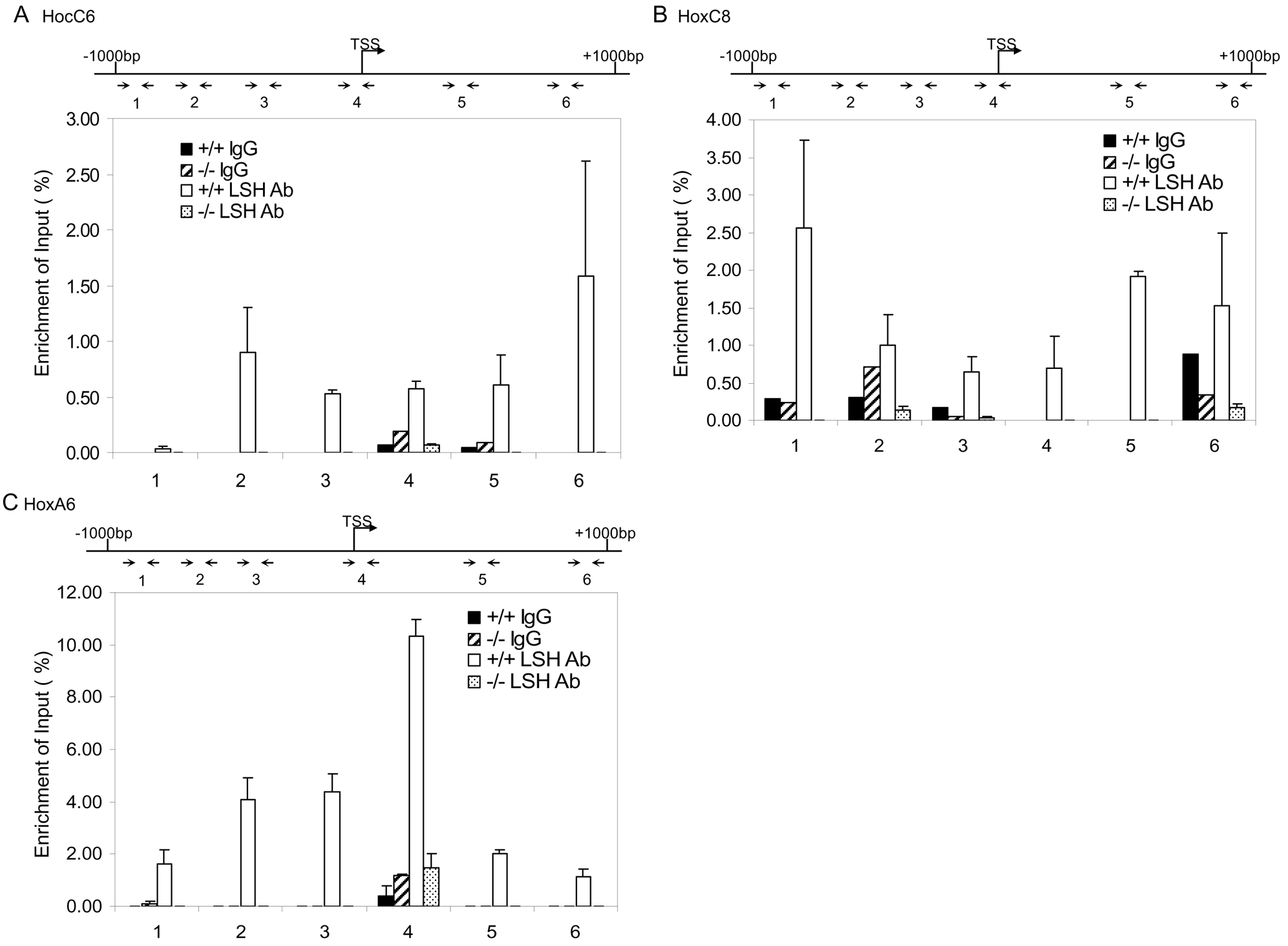

Supplement: Figure S1 — Lsh association with Hox genes. ChIP analysis using specific antibodies against Lsh (or control IgG) followed by PCR analysis to detect association of Lsh with promoter regions, TSS, and gene body regions of HoxC6 (A), HoxC8 (B) and HoxA6 (C) genes comparing Lsh+/+ and Lsh−/− MEFs. Enrichment is represented as a percentage of input. Error bars represent the standard deviation for the mean of three independent experiments. (0.50 MB TIF) [file pone.0009163.s001.tif]

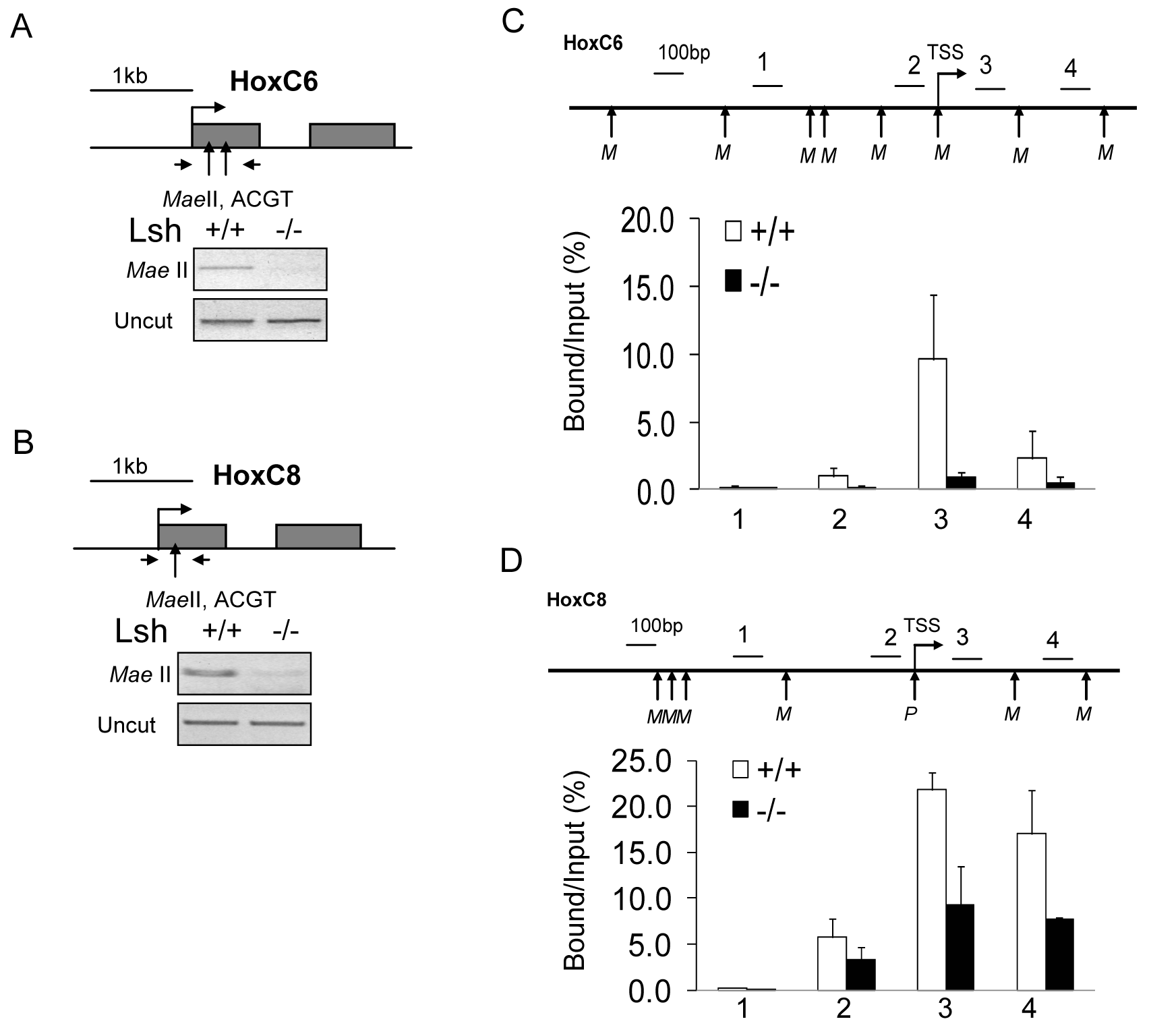

Supplement: Figure S2 — DNA methylation analysis at HoxC6 and HoxC8 genes. (A,B) Methylation sensitive PCR to determine DNA methylation level at HoxC6 (A) and HoxC8 (B) genes. The map on top illustrates the position of methylation-sensitive restriction enzyme (Mae II), as well as the primers used for the methylation-sensitive PCR analysis (Top). PCR analysis using genomic DNA derived from Lsh+/+ and Lsh−/− MEFs after digestion with Mae II. The undigested DNA served as a control for equal input of DNA (Uncut). (C,D) MeDIP analysis for detection of CpG methylation at the HoxC6 (C) or HoxC8 (D) genes. Region 1 (devoid of CpG sites) was amplified as negative control. Genomic DNA was digested with Mse I (C) or Mse I and Pst I (D), immunoprecipitated with anti-5-methylcytosine antibodies and specific Hox locations assessed by real-time PCR. Binding was expressed as a percentage of the input DNA. The bar graphs represent the mean of two independent MeDIP experiments. (0.39 MB TIF) [file pone.0009163.s002.tif]

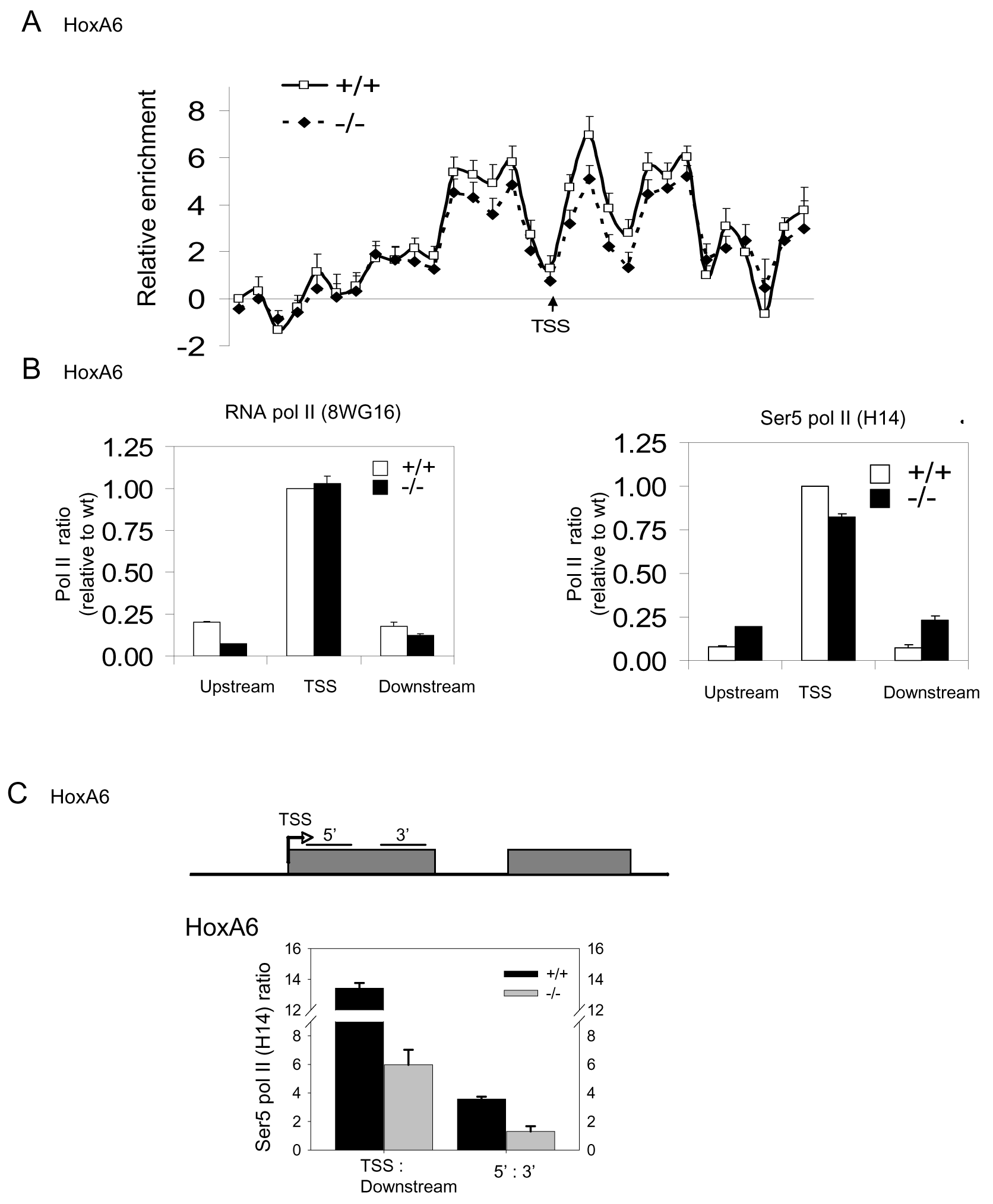

Supplement: Figure S3 — MNase protection profile at HoxA6. (A) The MNase protected profile of HoxA6 was determined by normalizing the amount of the MNase derived PCR products to those of the untreated DNA. (B) Enrichment of unphosphorylated Pol II (left) and Ser 5 Pol II (right) at HoxA6. (C) Ratio of Ser 5 Pol II ChIP signals at the TSS region over the downstream region of HoxA6 and the ratio of Ser 5 Pol II signal using a primer set located within exon1. (0.43 MB TIF) [file pone.0009163.s003.tif]

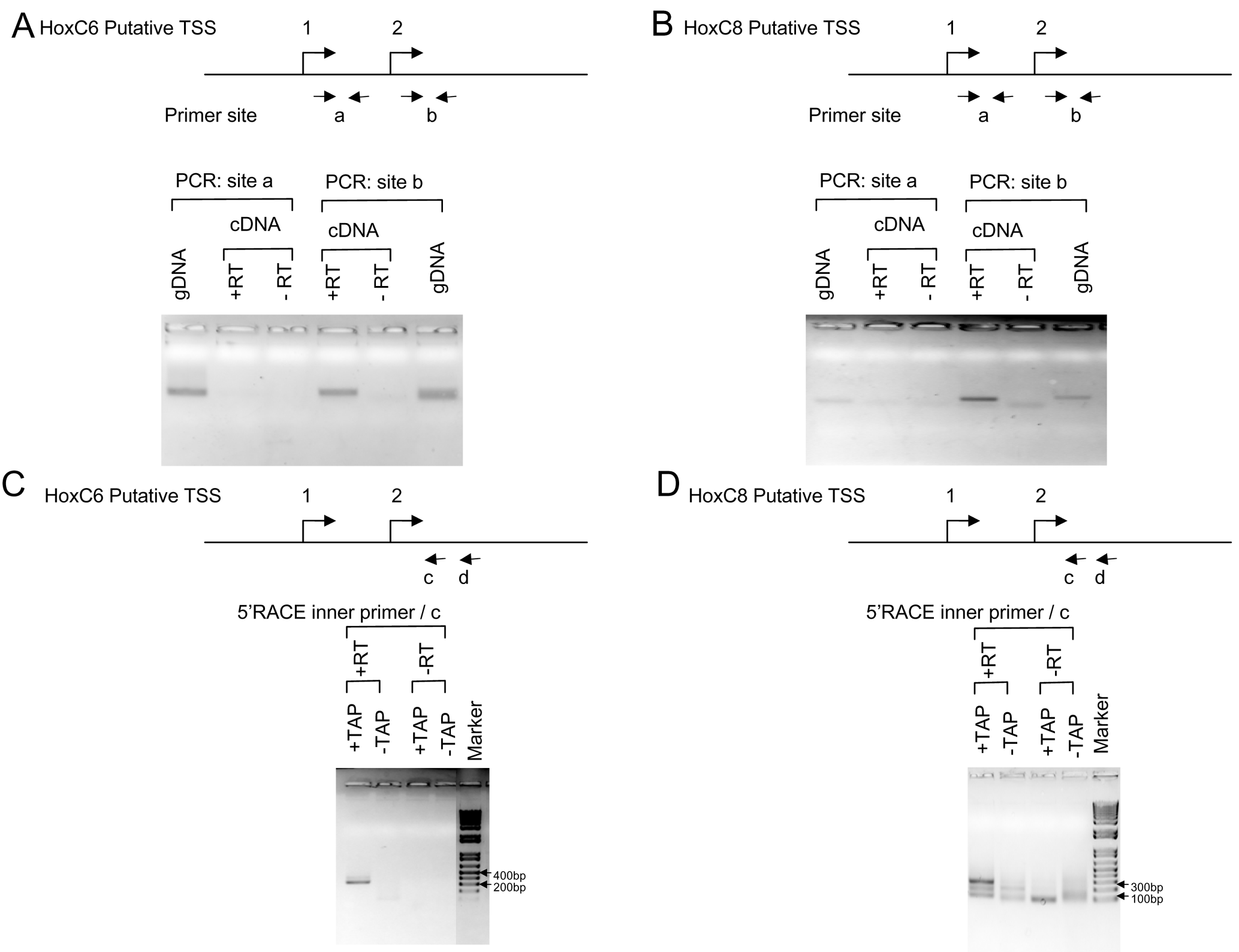

Supplement: Figure S4 — Identification of major transcriptional start sites (TSS) at HoxC6 and HoxC8 genes. (A). Identification of HoxC6 TSS using cDNA derived from Lsh−/− MEFs and primer specific for TSS region 1 and region 2. RT-PCR analysis suggest that regions 2 (as marked in Fig. 1 and Fig. 2 as TSS) is indeed the major TSS for HoxC6 since no signal was detected fro region 1. (B) Identification of HoxC8 TSS using cDNA derived from Lsh−/− MEFs and primer specific for TSS region 1. (C,D) Identification of HoxC6 (C) and HoxC8 (D) TSS using RLM-RACE. RNA is treated with tobacco acid pyrophosphatase (TAP) to remove the cap structure from the full-length mRNA leaving a 5′-monophosphate which is subsequently ligated to a synthetic RNA adaptor. After reverse transcription nested gene specific primers and adaptor specific primers are used for gene specific amplification. Results indicate a major amplification product about 320 bp for HoxC6 and 290 bp for HoxC8 confirming the preferential use of the predicted TSS for both genes. The major TSSs are reported in the Mouse Genome database (MGI) at chr15:102,839,993 for HoxC6 and at chr15:102,820,970 for HoxC8. (0.63 MB TIF) [file pone.0009163.s004.tif]

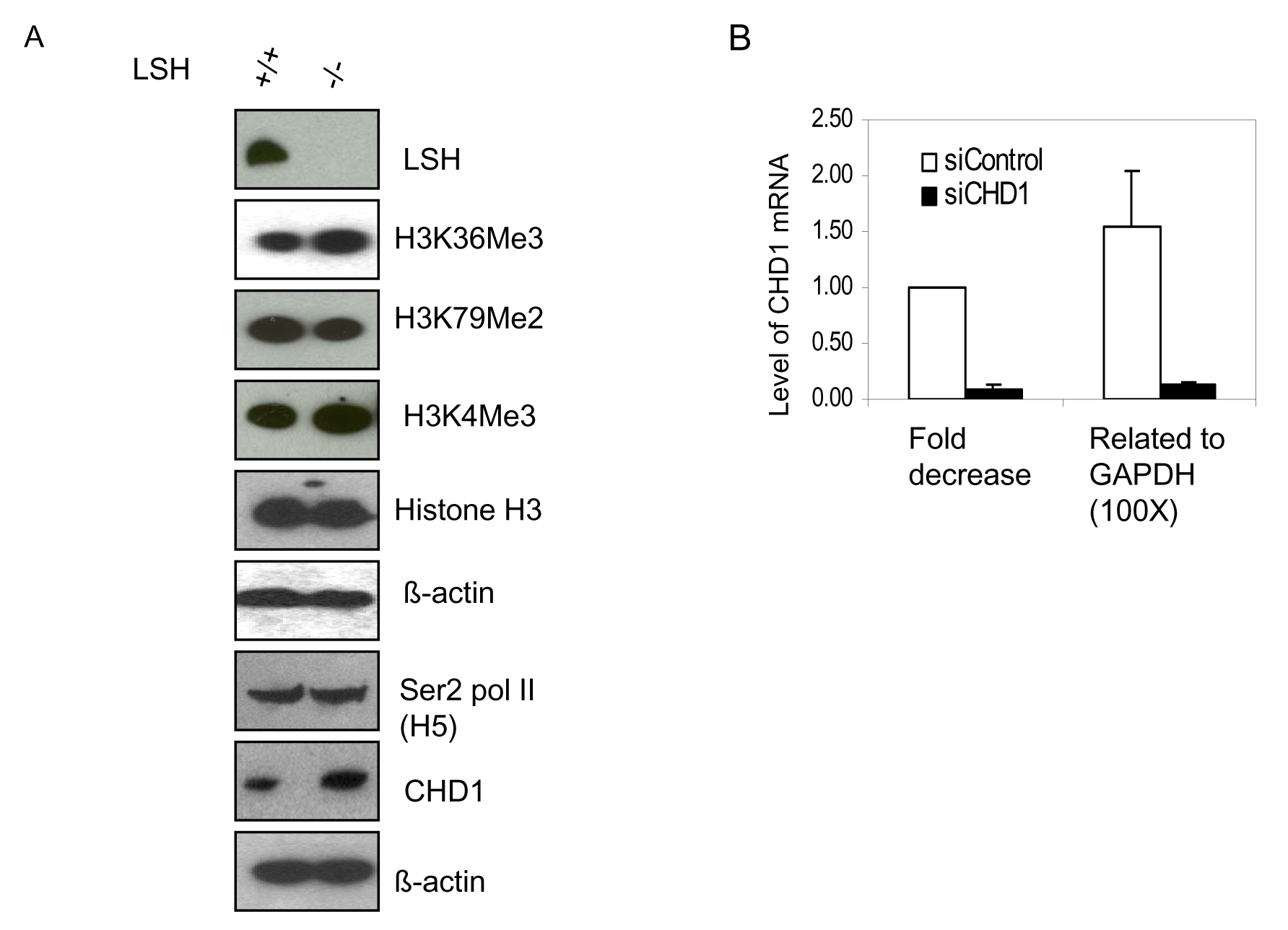

Supplement: Figure S5 — Protein analysis in the absence of Lsh. (A) Western analysis of extracts derived from Lsh+/+ and Lsh−/− MEFs using the indicated antibodies. (B) Real-time RT-PCR analysis for detection of Chd1 mRNA from Lsh−/− MEFs treated with control siRNA (siControl) or siRNA targeted against Chd1 (siChd1) for 48 hrs. (0.46 MB TIF) [file pone.0009163.s005.tif]

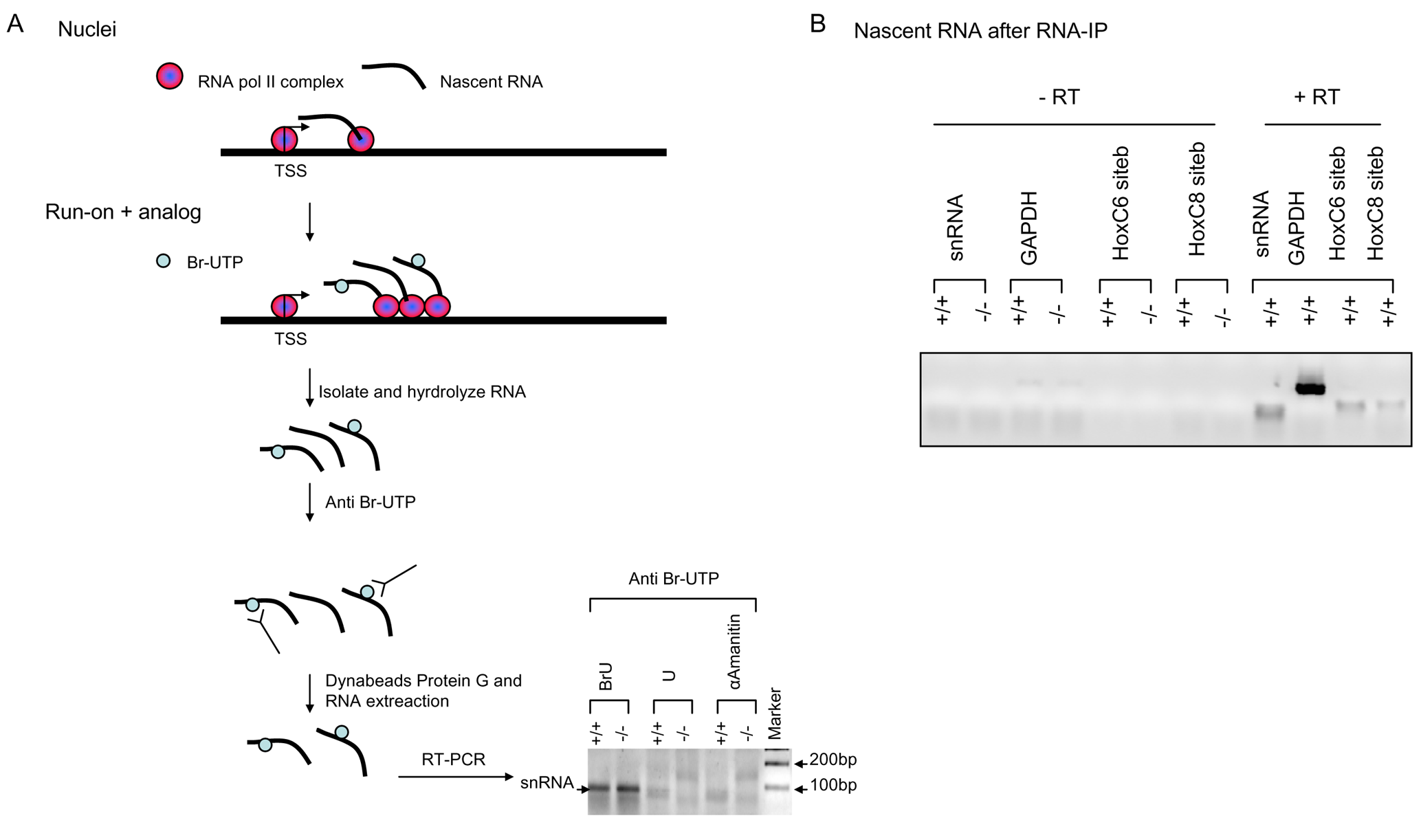

Supplement: Figure S6 — Nuclear run on assay. (A) Schematic diagram for the nuclear run-on assay based on the use of BrUTP. The nascent transcript snRNA detection was demonstrated by analyzing total RNA from Lsh+/+ and Lsh−/− MEFs labeled with BrU, Uridine (U) and BrU in the presence of α-amanitin. (B) PCR products with reverse transcriptase (+RT) or without reverse transcriptase (-RT) from nascent RNA that were immunoprecipitated by anti-Br-UTP antibody for detection of nascent RNA generated in the nuclear run on assay. The WT sample generated with RT served as positive control. Data indicate the purity of the nascent RNA used for the assay. (0.43 MB TIF) [file pone.0009163.s006.tif]

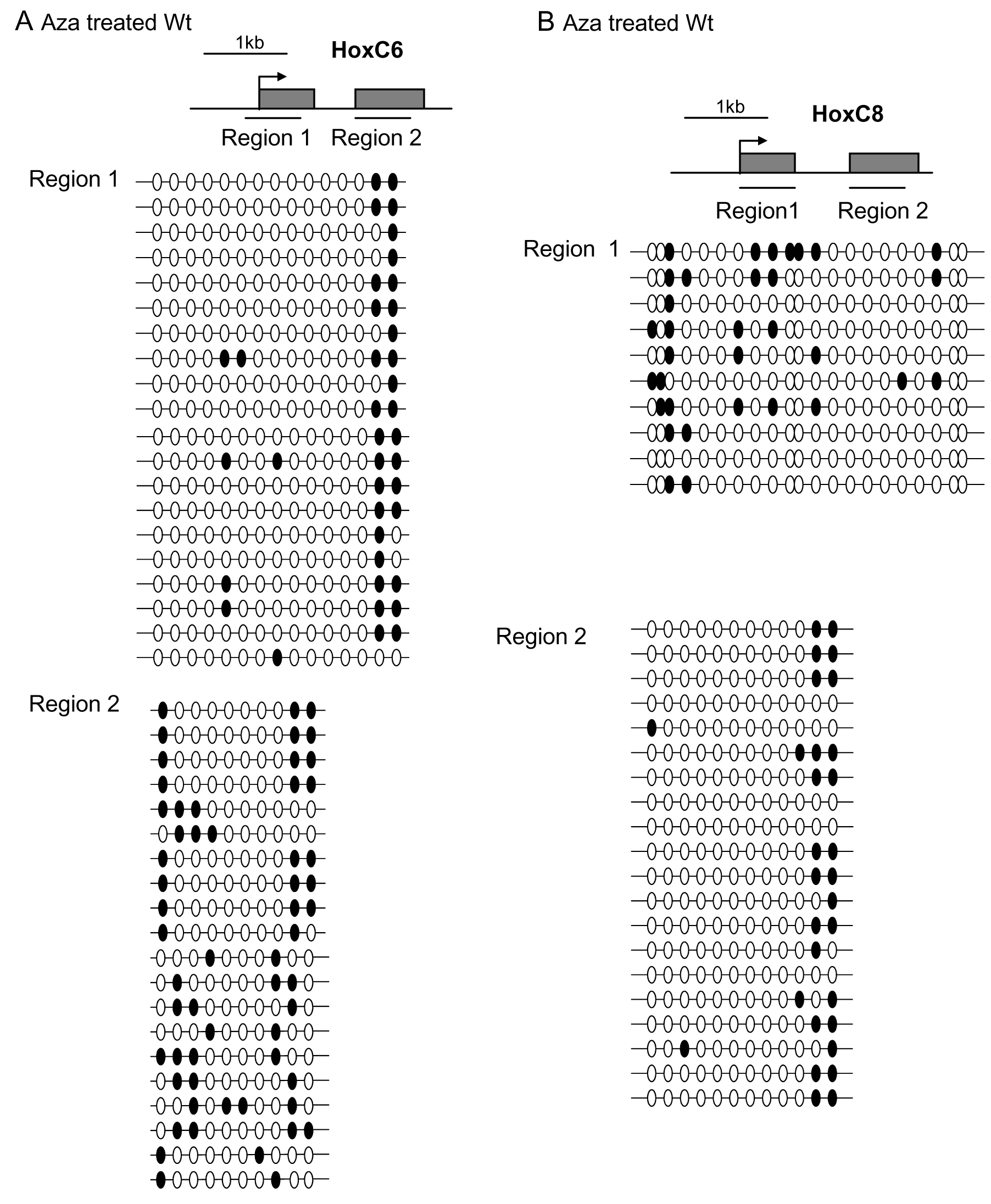

Supplement: Figure S7 — DNA hypomethylation after 5-Azacytidine treatment. Genomic DNA derived from Lsh+/+ MEFs before and after treatment of 5-Azacytidine was examined by bisulphite sequencing at region 1 (top) and region 2 (2nd exon, bottom) at the HoxC6 (A) and the HoxC8 (B) genes. (0.72 MB TIF) [file pone.0009163.s007.tif]

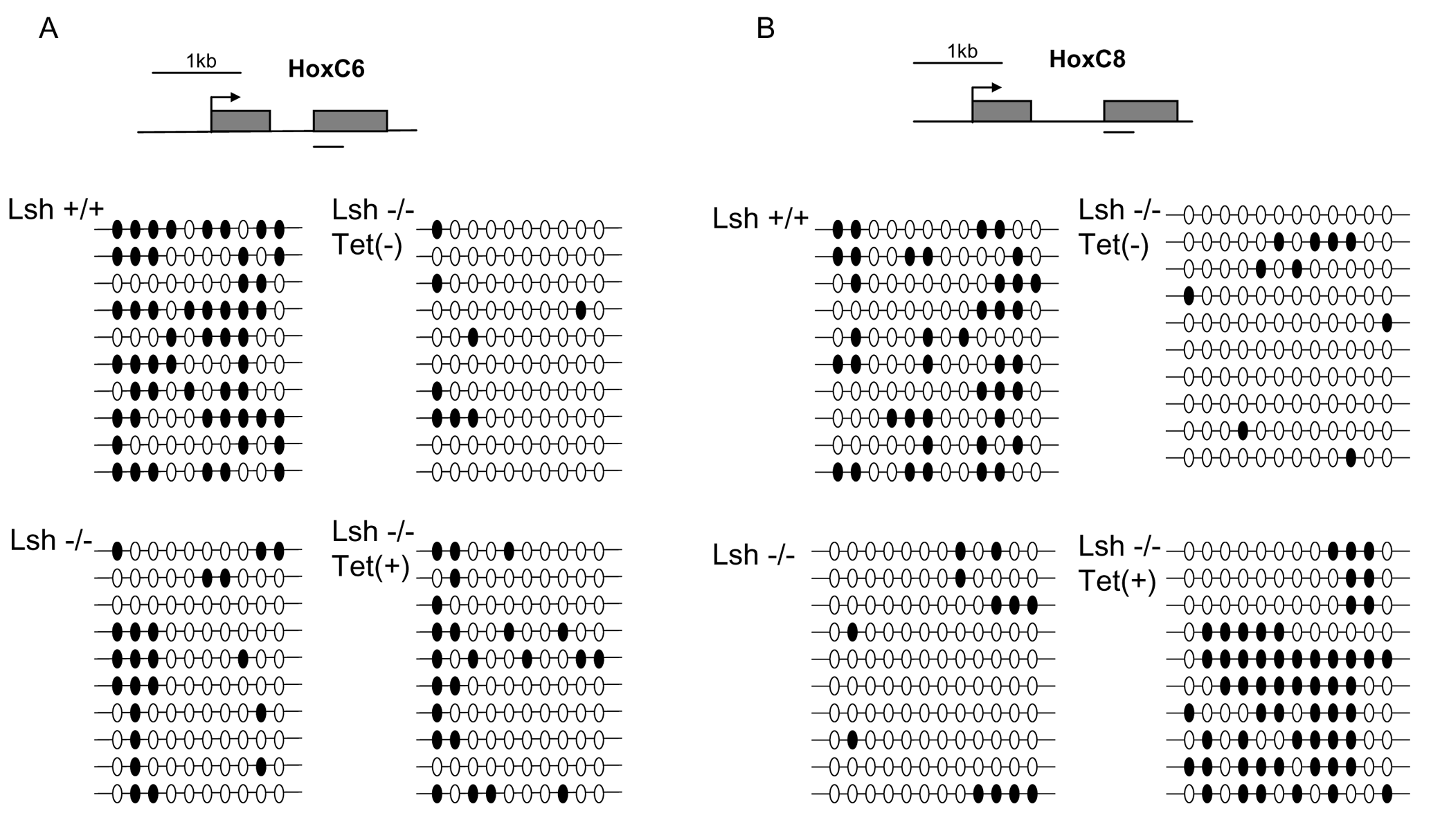

Supplement: Figure S8 — Re-introduction of Lsh in Lsh−/− MEFs. Genomic DNA derived from Lsh+/+ and Lsh−/− MEFs before and after 24 hours of tetracycline treatment to induce Lsh was examined by bisulphite sequencing at regions of the second exon at the HoxC6 (A) or HoxC8 (B) genes. Methylated CpGs are presented by black circles and unmethylated sites by open circles. (0.74 MB TIF) [file pone.0009163.s008.tif]

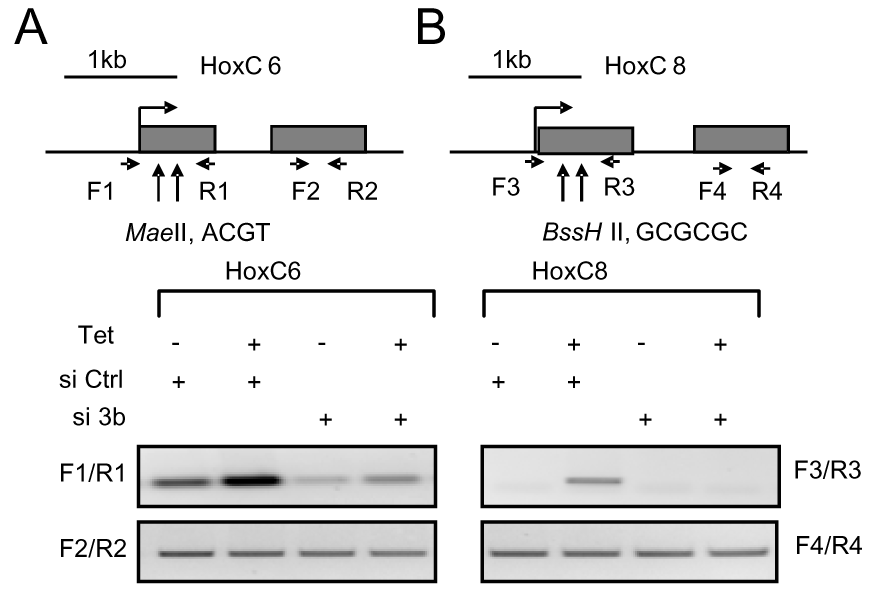

Supplement: Figure S9 — DNA hypomethylation after Dnmt3b depletion. Methylation sensitive PCR to determine methylation level at HoxC6 (A) and HoxC8 (B) after targeting by Dnmt3b siRNA or control siRNA before and after 24 hours of tetracycline treatment to induce Lsh in Lsh−/− cells. The map on top illustrates the position of methylation-sensitive restriction enzyme (Mae II in HoxC6 and BssH II), as well as the primers used for methylation-sensitive PCR analysis (Top). PCR analysis using genomic DNA derived from Lsh−/− MEFs after digestion with Mae II and BssH II. The digested DNA without enzyme sites served as a control for equal input of DNA (Bottom). (0.11 MB TIF) [file pone.0009163.s009.tif]

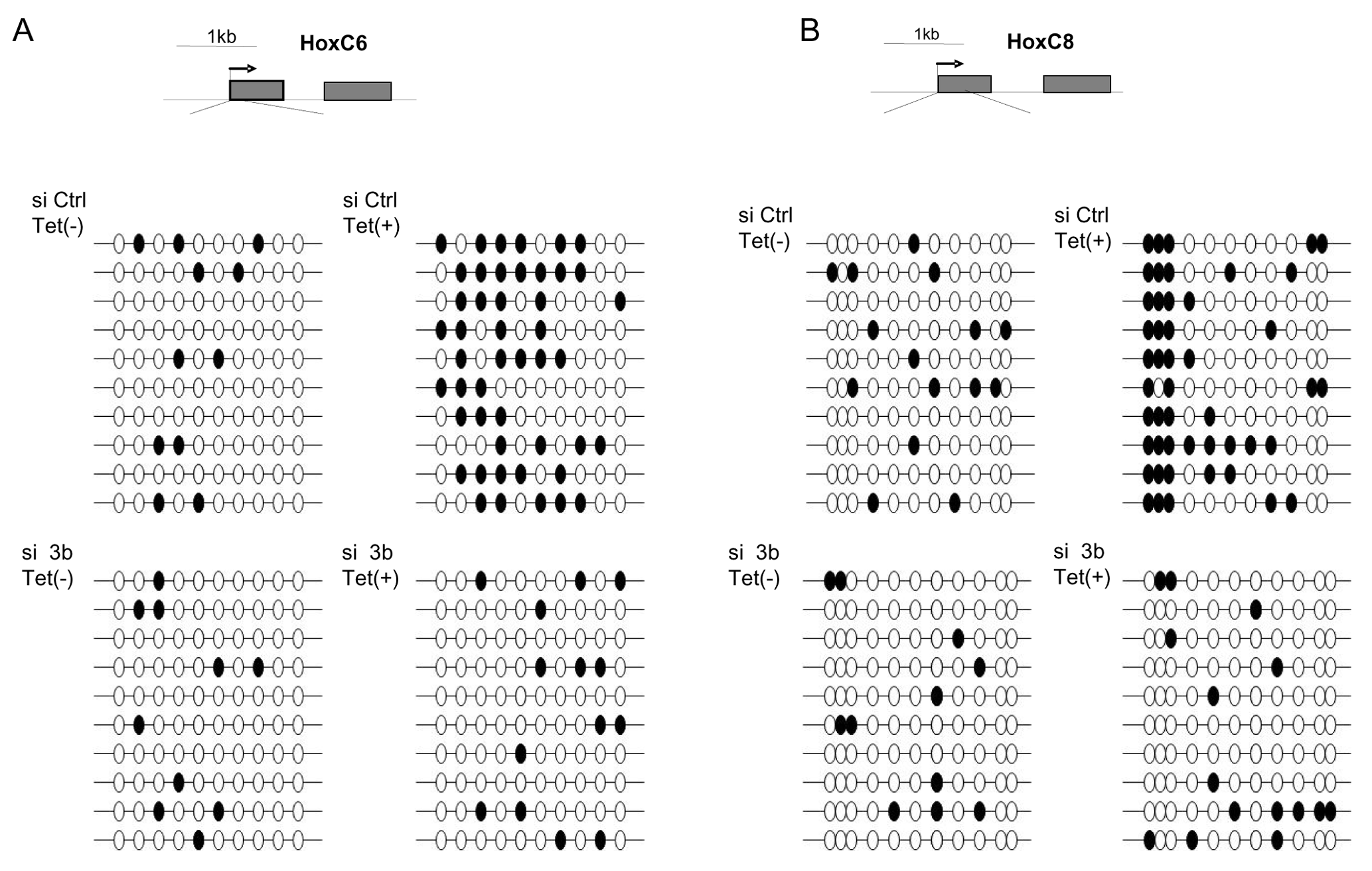

Supplement: Figure S10 — Dnmt3b is required for re-methylation at HoxC6 and HoxC8. Genomic DNA derived from Lsh−/− MEFs after targeting by Dnmt3b siRNA or control siRNA before and after 24 hours of tetracycline treatment to induce Lsh. HoxC6 (A) and the HoxC8 (B) genes were examined by bisulphite sequencing. Methylated CpGs are presented by black circles and unmethylated sites by open circles. (1.39 MB TIF) [file pone.0009163.s010.tif]
