# Supplementary material for: Lsh Mediated RNA Polymerase II Stalling at HoxC6 and HoxC8 Involves DNA Methylation
Source: PLoS One. 2010 Feb 11;5(2):e9163. doi: 10.1371/journal.pone.0009163 (PMC2820093; doi:10.1371/journal.pone.0009163)
Supplement: Table S1 — Primers for PCR, ChIP, Nuclear Run on and real-time PCR (0.16 MB DOC) [file pone.0009163.s011.doc]

|  | | | | |
| --- | --- | --- | --- | --- |
| **1. Primers for RT-PCR and real-time PCR to detect RNA levels in Hox genes** | | | | |
| Target | Primer ID | Sequence (5'→3') | |  |
| HoxC6 mRNA |  | F- ACCTTAGGACATAACACACAGA | | |
|  |  | R- ACTTCATGCGGCGGTTCTGGAA | | |
| HoxC8 mRNA |  | F- CCACGTCCAAGACTTCTTCCACCACGGC | | |
|  |  | R- CACTTCATCCTTCGATTCTGGAACC | | |
| HoxC9 mRNA |  | F- ACGTGGACTCGCTCATCTCT | | |
|  |  | R- GCCGTAAGGGTGATAGACCA | | |
| HoxA6 mRNA |  | F- CTGATAAAGACCTCAGTG | | |
|  |  | R- TCAGGTAGCGGTTGAAGTG | | |
| HoxA7 mRNA |  | F- CTCTGCAGTGACCTCGCCAAAG | | |
|  |  | R- CTTGTCAGCAGCTGTGGATTC | | |
| GAPDH mRNA | 1 | F- GGAGACAACCTGGTCCTCAG | | |
|  |  | R-ACCCAGAAGACTGTGGATGG | | |
|  | 2 | F-AACGACCCCTTCATTGAC | | |
|  |  | B-TCCACGACATACTCAGCAC | | |
| HoxC6 mRNA Real-time PCR | c | F-CCAGGACCAGAAAGCCAGTA | | |
|  |  | R-CCGAGTTAGGTAGCGGTTGA | | |
| HoxC8 mRNA Real-time PCR | c | F-CCTCCGCCAACACTAACAGT | | |
|  |  | R-CAAGGTCTGATACCGGCTGT | | |
| HoxA6 mRNA Real-time PCR |  | F-CTGATAAAGACCTCAGTG | | |
|  |  | B-TCAGGTAGCGGTTGAAGTG | | |
| HoxA7 mRNA Real-time PCR |  | F-CTCTGCAGTGACCTCGCCAAAG | | |
|  |  | B-CTTGTCAGCTGCTGTGGAAAC | | |
| CHD1 mRNA |  | F-GAGAAAGAGGCAACCGAGTG | | |
|  |  | B-TCTTGCCTGAGCCTGAAGAT | | |
| CHD1 mRNA Real-time PCR |  | F-GGGATGAGCTGCTTTCTCAG | | |
|  |  | B-TAGTCGCCGTCTTTGCTCTT | | |
| Dnmt3b |  | F-CCGGAAAATCACCAAGAAAA | | |
|  |  | B-CGAAGAAGAGCCTTCCTGTG | | |
| U2snRNA |  | F-GGAGTTGGAATAGGAGCTTGC | | |
|  |  | B-TGCACCGTTCCTGGAGGTAC | | |
| HoxC6 | a | F-ATAGCCCGACCAGGTAAAGG | | |
|  |  | B-ATCATAGGCGGTGGAATTGA | | |
|  | b | F-CCAGGACCAGAAAGCCAGTA | | |
|  |  | B-TCTAGGGAAGCCGGTCATAA | | |
| HoxC8 | a | F-GTGGATTGATGAACGCGAAT | | |
|  |  | B-CAGCTCTCTGCTCACTGTCG | | |
|  | b | F-CCTCCGCCAACACTAACAGT | | |
|  |  | B-CAAGGGGGAAAGAGAAAAGG | | |
|  |  |  |  |  |
| **2. Methylation-sensitive PCR** | |  |  |  |
| HoxC6 | 100F | F-ATAGCCCGACCAGGTAAAGG | | |
|  | 200+1B | B-TTTGTCTGCAGTTTGAGAGCA | | |
| HoxC8 | 300F | F-GGGTTTTCATGTACCCAGCA | | |
|  | 500B | B-CTCTTGCTGAGCCCCATAAA | | |
|  |  |  |  |  |
| **3. MeDIP** |  |  |  |  |
| HoxC6 | 1 | F-GCACAAGGTTAGGAGGCAAA | | |
|  |  | B-AGGCAGTGTGGGAGACAAAC | | |
|  | 2 | F-CAACAGAAGCAGAAGCGATTT | | |
|  |  | B-TCCAATCCAGGACAGACAAA | | |
|  | 3 | F-ATTCGCCACAGGAGAATGTC | | |
|  |  | B-TTTGTCTGCAGTTTGAGAGCA | | |
|  | 4 | F-GGAGCAGAGGGGGTAGAAAC | | |
|  |  | B-CCACTTCTTTGCCCTTTCCT | | |
|  | -900 | F-TTTAAAAAGAATCTCAGTCTCTCTCTC | | |
|  |  | B-CTACCTTTTGGGGAAACTTTTAT | | |
|  | -100 | F-CAACAGAAGCAGAAGCGATTT | | |
|  |  | B-TCCAATCCAGGACAGACAAA | | |
|  | TSS | F-TTTGTCTGTCCTGGATTGGA | | |
|  |  | B-CCTTTACCTGGTCGGGCTAT | | |
|  | 200 | F-ACCGCCTATGATCCAGTGAC | | |
|  |  | B-GCTGGAACTGAACACGACATT | | |
|  |  |  |  |  |
| HoxC8 | 1 | F-GGCATTTCCTAAGGGATGGT | | |
|  |  | B-GTTTGTGTGCCCAGGTTTCT | | |
|  | 2 | F-CAGCCCTGAACCCCCTCT | | |
|  |  | B-CGAGCGAGCAAGAGAGAGA | | |
|  | 3 | F-CCCTCTCCCTCTCCCTCT | | |
|  |  | B-CGTTCATCAATCCACGACAT | | |
|  | 4 | F-CTCAGAGCGTGGGCAGAAG | | |
|  |  | B-AGCATGGGTTCTGCTGGTAG | | |
|  | -900 | F-GGCATTTCCTAAGGGATGGT | | |
|  |  | B-GTTTGTGTGCCCAGGTTTCT | | |
|  | -100 | F-CAGCCCTGAACCCCCTCT | | |
|  |  | B-CGAGCGAGCAAGAGAGAGA | | |
|  | TSS | F-CCCTCTCCCTCTCCCTCT | | |
|  |  | B-CGTTCATCAATCCACGACAT | | |
|  | 200 | F-AGGGGAGTTTCGGGGATA | | |
|  |  | B-CCGCCTTTGTATTTGGAAAA | | |
|  |  |  |  |  |
| **4. Bisulfite sequencing** |  |  |  |  |
| HoxC6 | -2 | F-TTTATATATTGTTTGTTTTTGGGTT | | |
|  |  | B-CAATCACCACTAAAATCTCCATTAC | | |
|  | -1 | F-GAAATGGGTTTGAAAATAAGGATTAT | | |
|  |  | B-AATAAAAATTCATCCCTTTACCTTTAC | | |
|  | TSS | F-TGAAAATAAATATTAAAGAAATTATAGTT | | |
|  |  | B-AAAAAAATTAAATCCATAATCATAC | | |
|  | 1 | F-TTTAGTTTTGAGTAGGGTAGGATTG | | |
|  |  | B-TTAAAAAACTCCTCCACCTTCTATC | | |
|  | 2nd Exon | F-ATTGGAGAAGGAATTTTATTTTAAT | | |
|  |  | B-ATCCAAAACTAACTTTTAAATCCAC | | |
| HoxC8 | -3 | F-GAAAGGTTTTGGTTTTTGTTAGATT | | |
|  |  | B-AATTTCCCCTTTAAAAACTTATCTC | | |
|  | TSS | F-TTTGGGTTGTTAGAGGAAAGAGTTA | | |
|  |  | B-ATAAAAACTCATACTAAATACATAAAAACC | | |
|  | 1 | F-AATTTTTTGTTTTTTAAATATAAAGG | | |
|  |  | B-CAAACACAATATCTCAAACACCC | | |
|  | -1 | F-GAAAGAAAAGGGGGAATATTTTTA | | |
|  |  | B-TAACTCTTTCCTCTAACAACCCAAA | | |
|  | 2nd Exon | F-ATGGAAATTTTTTTTGTTTTTGTTT | | |
|  |  | B-ACCCCAAACAATTTATCCTTATTATT | | |
|  |  |  |  |  |
| **5. ChIP** |  |  |  |  |
| HoxC6 upstream | 1 | F-GCACAAGGTTAGGAGGCAAA | | |
|  |  | B-AGGCAGTGTGGGAGACAAAC | | |
|  | 2 | F-GCCACAGCACAGCAAAGTTA | | |
|  |  | B-GGTGGGGTGAAGGTCTATGA | | |
| HoxC6 TSS | 1 | F-ATAGCCCGACCAGGTAAAGG | | |
|  |  | B-ATCATAGGCGGTGGAATTGA | | |
|  | 2 | F-CAGGTAAAGGCAAAGGGATG | | |
|  |  | B-CAACGGCTGCTCCATAGGTC | | |
| HoxC6 Downstream | 1 | F-CATGCTCTCAAACTGCAGACA | | |
|  |  | B-TGCATCCAGGGGTAAATCTG | | |
|  | 2 | F-GGAGCTGAGGGGGTAGAAAC | | |
|  |  | B-CCACTTCTTTGCCCTTTCCT | | |
| HoxC6 3'UTR | 1 | F-CTCTGTCTCCGGGTTCTCC | | |
|  |  | B-CGGGTCAGGAGTCTCAGAAC | | |
|  | 2 | F-ATGCAAGAAAACGAGCCTGA | | |
|  |  | B-CTCAGCCAGGTTTCACAACA | | |
|  |  |  |  |  |
| HoxC8 Upstream | 1 | F-GAGATTGTGCAGGCAGAAGG | | |
|  |  | B-AGCCTGCAATCCAAAAGATG | | |
|  | 2 | F-TCCTTGGAGAGGTTGCTTTC | | |
|  |  | B-AGGTCTGGAGCTGCTCTGTC | | |
| HoxC8 TSS | 1 | F-GTGGACTTGATGAACGCGAAT | | |
|  |  | B-CAGCTCTCTGCTCACTGTCG | | |
|  | 2 | F-CGACAGTGAGCAGAGAGCTG | | |
|  |  | B-CGGGTATCCCCGAAACTC | | |
| HoxC8 Downstream | 1 | F-GGAACCGGCCTATTACGACT | | |
|  |  | B-CGTGGTGGAAGAAGTCTTGG | | |
|  | 2 | F-TCCTTTTTGTCTGCCCTAGC | | |
|  |  | B-CAGCCAAGCCTTTCAATCTC | | |
| HoxC8 3'UTR | 1 | F-CGAGATGAGGAGAAGGTGGA | | |
|  |  | B-GGGGGCTGATTTTCTCTCTC | | |
|  | 2 | F-CCCTCTGAGGCTCTTTCCTT | | |
|  |  | B-CGGGTCAGGAGTCTCAGAAC | | |
